# A multi-analytical study of the Montelirio beaded attires: Marine resources, sumptuary crafts, and female power in copper age Iberia

**DOI:** 10.1126/sciadv.adp1917

**Published:** 2025-01-29

**Authors:** Leonardo García Sanjuán, Samuel Ramírez-Cruzado, Marta Díaz-Guardamino, José Antonio Lozano Rodríguez, Teodosio Donaire Romero, José Ángel Afonso Vargas, Carlos Rodríguez-Rellán, Verónica Balsera Nieto, Luis M. Cáceres Puro, David W. Wheatley, Timothy Earle, Marta Cintas-Peña, Juan Manuel Vargas Jiménez, Álvaro Fernández Flores, Miriam Luciañez Triviño, Juan Cárdenas-Párraga, María Martínez Merino, Fernando Muñiz Guinea

**Affiliations:** ^1^ Department of Prehistory and Archaeology, University of Seville, Seville, Spain.; ^2^ Department of Archaeology, Durham University, Durham, UK.; ^3^ Canary Islands Oceanographic Center (COC), Spanish Institute of Oceanography (IEO), Spanish Research Council (CSIC), Santa Cruz de Tenerife, Spain.; ^4^ Department of History and Philosophy, Area of Prehistory, University of Alcalá (UAH), Alcalá de Henares, Spain.; ^5^ Department of Earth Sciences, University of Huelva, Huelva, Spain.; ^6^ Department Animal Biology, Edaphology and Geology, University of La Laguna, Santa Cruz de Tenerife, Spain.; ^7^ Department of Prehistory and Archaeology, University of Granada, Granada, Spain.; ^8^ Instituto de la Juventud, Ministerio de Derechos Sociales y Agenda 2030, Madrid, Spain.; ^9^ Department of Archaeology, University of Southampton, Southampton, UK.; ^10^ Department of Anthropology, Northwestern University, Evanston, IL, USA.; ^11^ Servicio de Arqueología, Ayuntamiento de Valencina de la Concepción, Valencina de la Concepción, Seville, Spain.; ^12^ Arqueología y Gestion S.L.L., Seville, Spain.; ^13^ Conservation/Restoration Section, Department of Painting, University of the Basque Country UPV/EHU, Vizcaya, Spain.; ^14^ Department of Crystallography, Mineralogy and Agricultural Chemistry, Faculty of Chemistry, University of Seville, Seville, Spain.; ^15^ MUGUS, Museum of Geology of the University of Seville, Seville, Spain.

## Abstract

Excellent indicators of technology, social organization, exchange patterns, and even beliefs, beads are a topic of research in their own right. Findings made between 2010 and 2011 at the Montelirio tholos burial, part of the Valencina Copper Age mega-site, in south-western Spain, revealed what amounts to the largest single-burial ever-documented assemblage of beads. Furthermore, the Montelirio beads were part of unparalleled beaded attires worn by some of the people buried in the grave, mostly females. A multi-analytical study undertaken over the past 5 years—including a meticulous quantification of the collection, the characterization of the raw materials, radiocarbon dating and chronometric statistical modeling, morphometric analysis, phytolith analysis, experimental work and contextual analysis—reveals several previously unidentified aspects of these remarkable creations. This includes the role of the attires as sumptuary attributes heavily loaded of symbolism, used by a selected group of women of high social significance.

## Teaser

Beaded attires made ~2800 BC with ~270,000 shell beads were worn by a select group of prominent women.

## INTRODUCTION

**Fig. 1.**
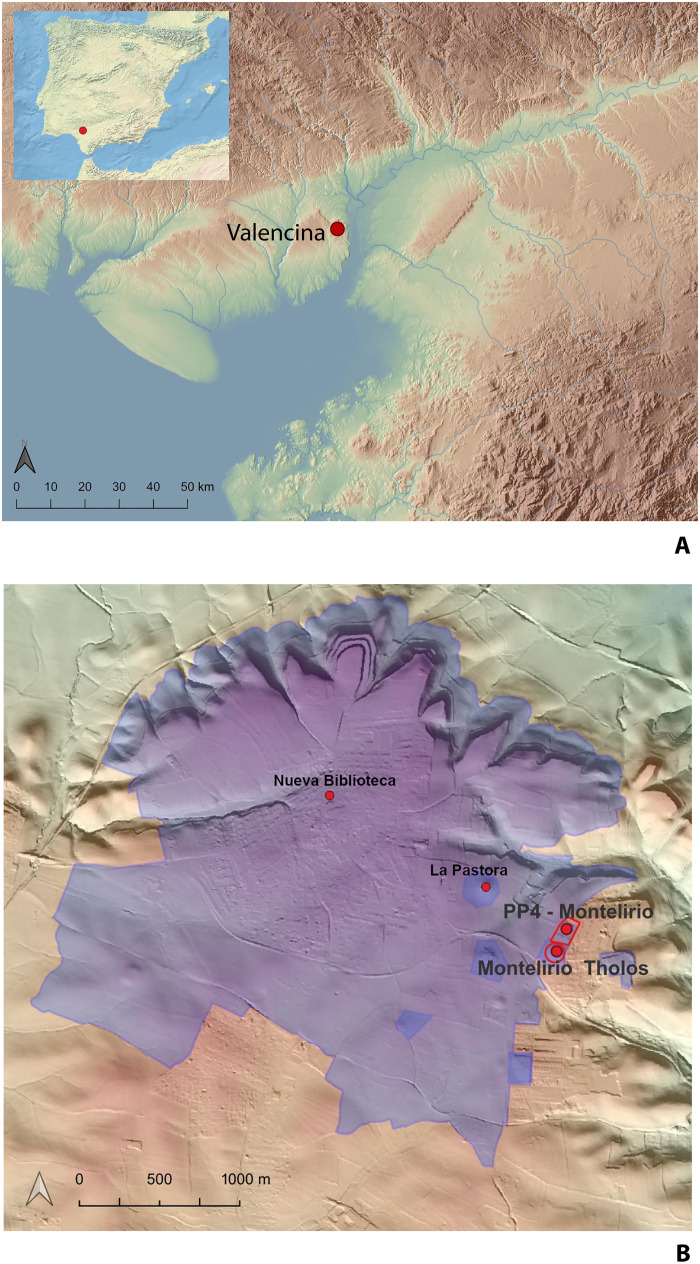
Location of the Valencina Copper Age mega-site in the Iberian Peninsula and the Montelirio tholos within the mega-site. (**A**) Location of Valencina in the Iberian Peninsula and the Spanish south-west, showing middle Holocene coastline. (**B**) Location of Montelirio and PP4-Montelirio Sector (with Structure 10.042–10.049) within the Valencina de la Concepción-Castilleja de Guzman mega-site (highlighted in purple). The map base is the nation-wide Lidar coverage (0.5 points/m^2^) issued by the Spanish National Aerial Ortophotography Plan and freely available online (https://pnoa.ign.es/pnoa-lidar/presentacion). Design: F. Sánchez Díaz.

Here, we study a large collection of discoidal perforated beads from the Montelirio tholos tomb and neighboring Structure 10.042–10.049 (also referred to as “The Ivory Lady” grave). Since they were excavated between 2007 and 2010 and then studied in publications including a monograph (*1*) and several papers [(*2*–*7*), etc.] these two tombs have, in barely a decade, become a paradigm of some of the main features associated with the Iberian Copper Age: economic intensification, emerging social complexity, supraregional connectivity, sophisticated monumentality, lavish material culture, and artistic exuberance. Both tombs are part of the Valencina de la Concepción-Castilleja de Guzmán (henceforth Valencina) mega-site, which, located in south-westen (SW) Spain (Fig. 1) and spreading over an area of c. 450 ha in the northern El Aljarafe plateau, barely 6 km from the city center of modern-day Sevilla, has itself become a major reference for the study of third millennium BC Europe (*8*–*11*). Over the past two decades, diverging interpretations have been put forward to interpret Valencina as a site, including it being a permanently occupied village acting as seat of a centralized state-level polity, a low-density settlement with shifting foci of occupation, a central place used for seasonal or periodical gatherings involving people living in the surrounding region (and further afield), a “cemetery,” or any combination of those [see (*2*) and (*8*) for a synthesis of this problem and further readings].

One chapter in the Montelirio monograph (*12*) offered a preliminary study of the remarkable beaded attires of this tomb. Here, a much-expanded version of that study is presented based on a multidisciplinary approach that includes completely new elements such as quantification, biogeological characterization, morphometric analysis, radiocarbon dating and modeling, archaeobotanical analysis, experimental work, and some theoretical consideration.

The main specific aims of this study are: (i) to quantify the bead assemblage reliably in order to understand the labor investment inherent to it and compare it with other major collections of beads, world-wide; (ii) to establish the chronology of the beads to understand their synchronicity (or otherwise) with the people buried in the tomb; (iii) to characterize the raw materials involved in the making of the beads and attires; and (iv) to understand the technical process involved through experimental archeology and phytolith analysis (intended to identify possible traces of the fibers the beads were weaved with). In general, in this work, we ask how and when these garments were made, what social and cultural context gave rise to these exceptional attires, and what are the implications of the technical aspects of these garments for our understanding of Copper-Age society.

## RESULTS

### Quantification

**Fig. 2.**
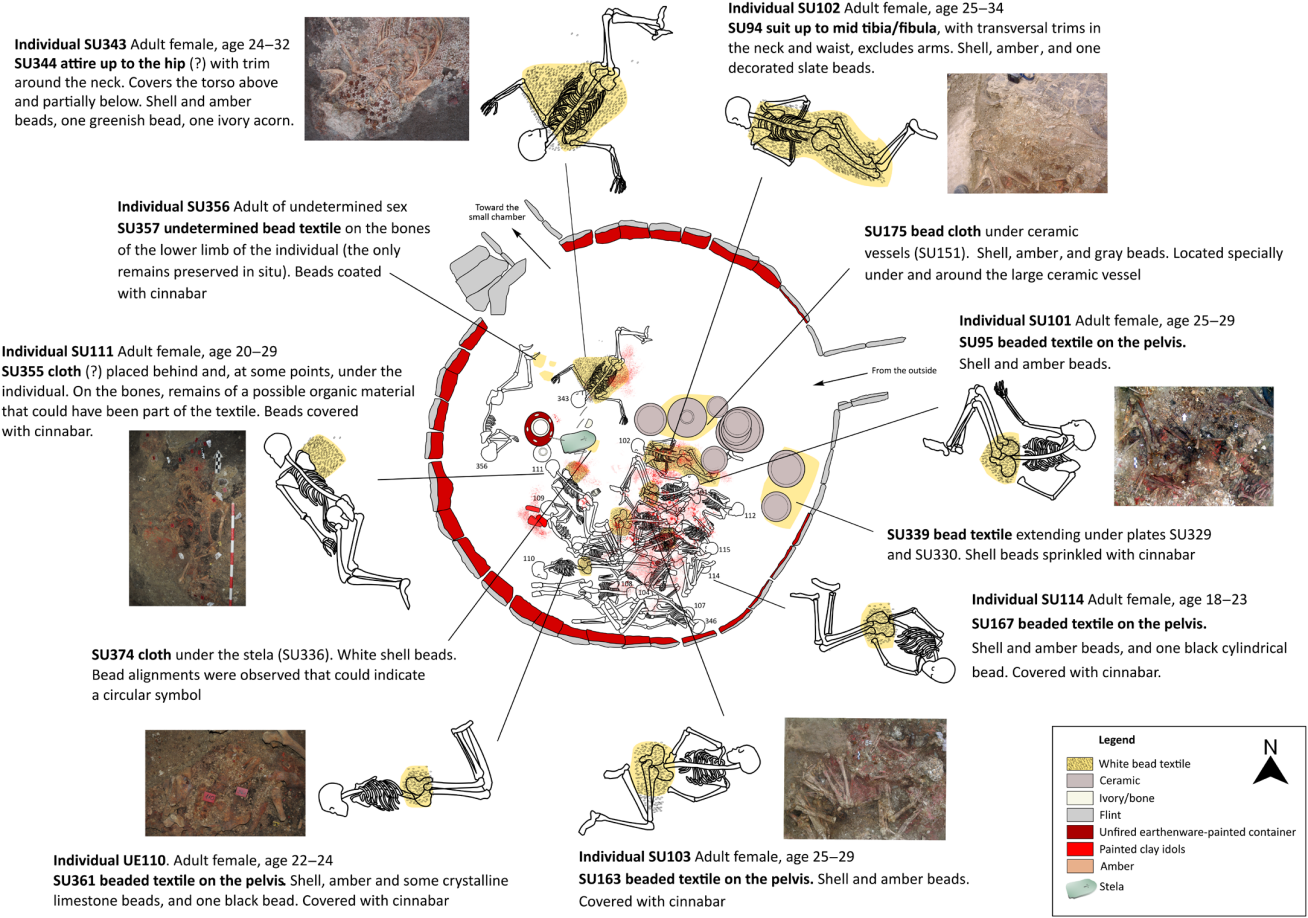
Distribution of the attires within the LC of Montelirio. Design: M. Luciañez Triviño and Á. Fernández Flores [based on figure 3 in (*12*)].

**Fig. 3.**
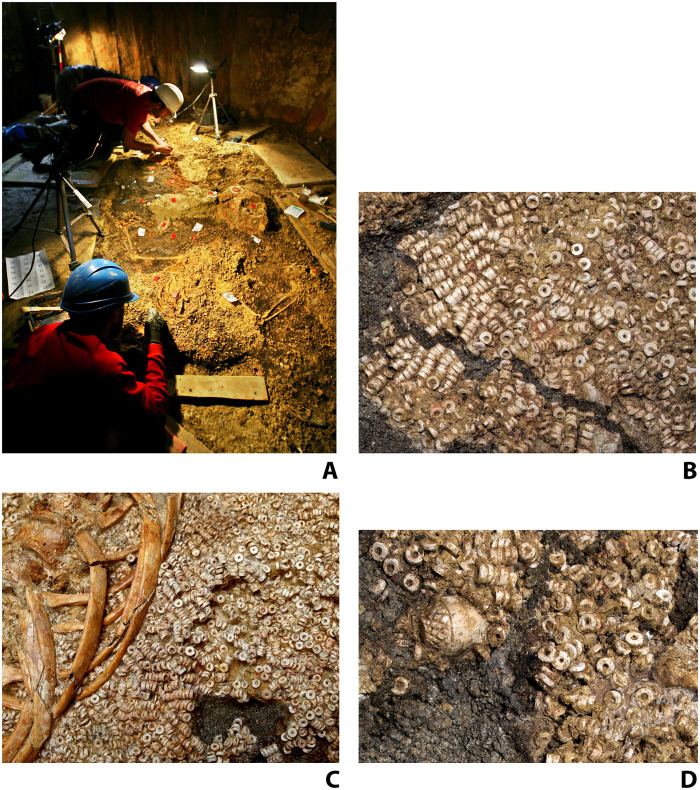
Montelirio beads in situ. (**A**) Excavation process of Individual 343. (**B**) Detail of the threaded beads recorded underneath Individual UE 343 (belonging to attire UE 344). (**C**) Detail of the threaded beads on attire UE 344 next to the bones of Individual 343. (**D**) Detail of the ivory acorn integrated in the fabric of attire UE 344, belonging to Individual UE 343. Photographs: (A) by A. Acedo García; [(B) to (D)] by D.W.W. A three-dimensional (3D) model of individual EU343 is available at https://skfb.ly/o7oWO. 3D model by M.D.-G. with photographs taken by D.W.W.

**Fig. 4.**
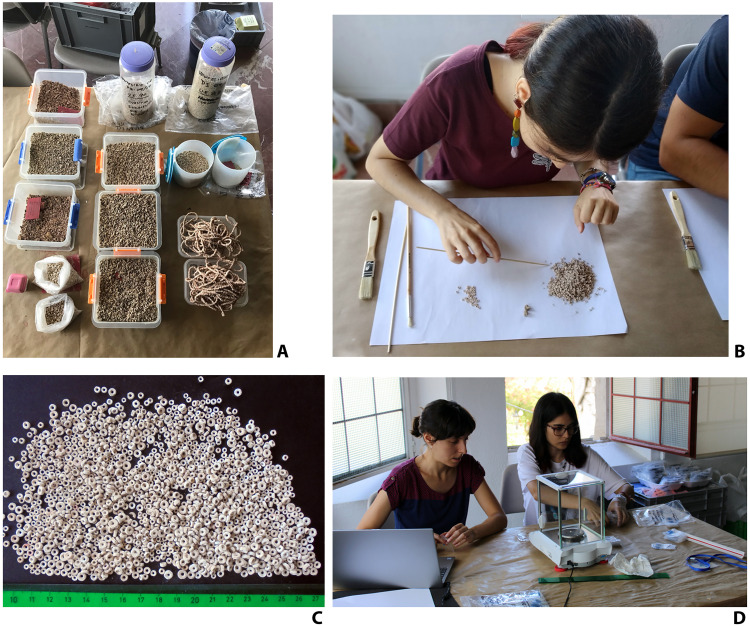
Cleaning and weighing of the beads in the Sevilla Archaeology Museum in July–August 2019. (**A**) Plastic containers containing the beads. (**B**) Manual cleaning of the beads after sieving. (**C**) Example of manually cleaned beads. (**D**) Weighing of the beads. Photographs: L.G.S.

The location of the beaded attires within the large chamber (LC) of Montelirio and the individuals who wore them, mostly females (*13*), are shown in Figs. 2 and 3. Basically, three types of textiles were identified: full body tunics (worn only by individuals UE343 and UE102), skirts, and cloths of undetermined shape. The excavation involved a painstaking process by which the attires were individualized and, in some cases, consolidated in situ. In Structure 10.042–10.049, located barely 100-m north of Montelirio, more than 2000 beads were found scattered in the corridor and the first chamber (which was largely destroyed). A small group of 90 beads were found in the upper level of the second chamber (Structure 10.049), in connection with a rock crystal blade dagger, of whose ivory handle the beads are believed to have been a decoration (*4*). As it was presented to us in the Sevilla Archaeology Museum in July 2019, the Montelirio assemblage of beads was stored in 51 containers (Fig. 4A). The beads were heavily mixed with soil from the infill of the tomb. Therefore, to quantify the material, the first job was to remove the soil mixed with the beads. A description of the procedure used is provided in Materials and Methods. Work involved seven people and approximately 651 human hours over 3 weeks.

**Fig. 5.**
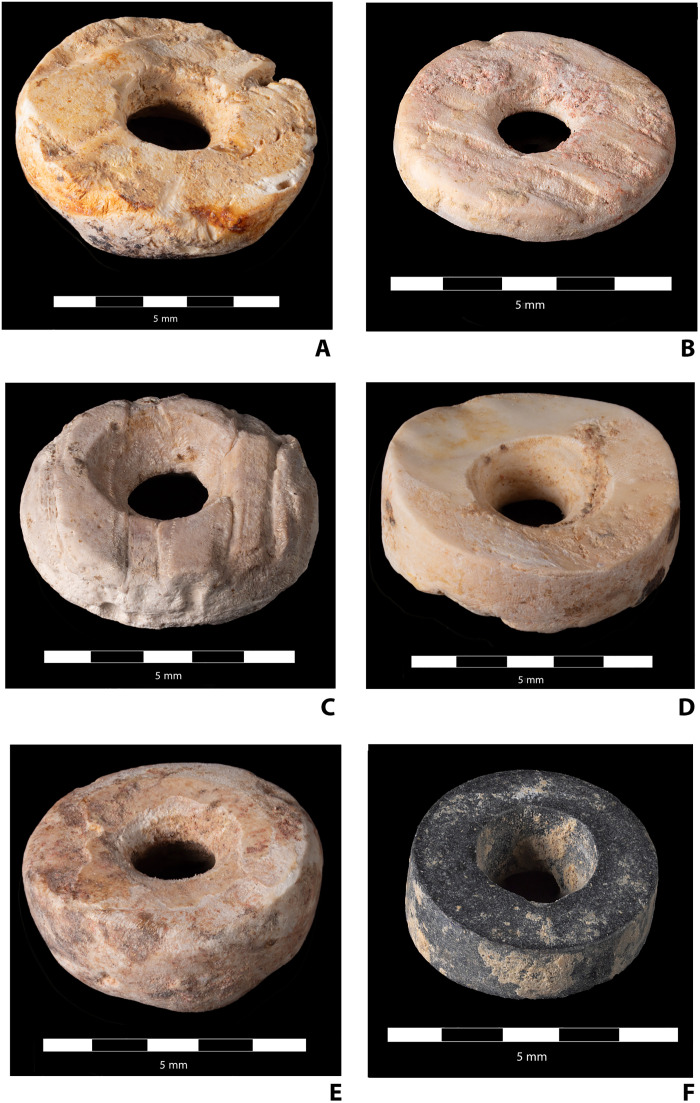
High-resolution photographs of selected beads. (**A**) Bead UE94, INV89, IND102, M020 (marine shell). (**B**) Bead IND101-102, UE94-95, M01 (marine shell). (**C**) Bead UE374 (cloth) and M051 (marine shell). (**D**) UE94, INV90, IND102, and M055 (marine shell). (**E**) Bead UE105, INV3, IND105, and M079 (marine shell). (**F**) Bead LRTII, E1, and UE53 (chloritoid schist). The photos are focus-stacked composites taken with a Canon R5 with MPE 65-mm f2.8 macro lens set at f5.6. The same ×3 magnification was maintained for every photograph. Lighting was achieved with two LED panels, and both camera and lights set to 5200 K to ensure consistent color representation. Camera was mounted on a computer-controlled, motorized macro rail (Cognisys Stackshot 3X), and raw format images were taken at 0.2-mm intervals resulting in a stack of 25 to 35 images for each bead. These were then combined using HeliconSoft “Helicon Focus” software, method B (depth map setting with radius = 8 and smoothing = 4; these parameters established by experimentation), to produce the resulting final images. Scale was established by photographing physical scales with identical settings and then adding the scale graphic to each image using Photoshop, at which time minor additional corrections of the background were also undertaken to remove dust, etc. Photographs by D.W.W.

Once the cleaning was completed (Figs. 4, B and C; 5; and 6), samples of the soil attached to the beads were taken for phytolith analysis (see below), and the materials were weighed, resulting in 6.93 kg of dirt and stones (which were removed from the beads) and 11.13 kg of beads, including 7.71 kg from LC and 3.42 kg from the small chamber (SC), as well as 0.46 kg from the tomb of The Ivory Lady. A nontrivial amount of the beads found in Montelirio (particularly those of the attire worn by Individual UE 343, individual 111, and cloth 339) were consolidated in situ during the excavation and then transported as blocks to the museum (a 3D model of individual EU343 is available at https://skfb.ly/o7oWO). For this reason, they could not be weighed individually. Therefore, while 11.3 kg of beads was cleaned, weighed, and recorded in the museum, a more accurate overall figure for the total Montelirio assemblage would be between 13 and 15 kg.

**Fig. 6.**
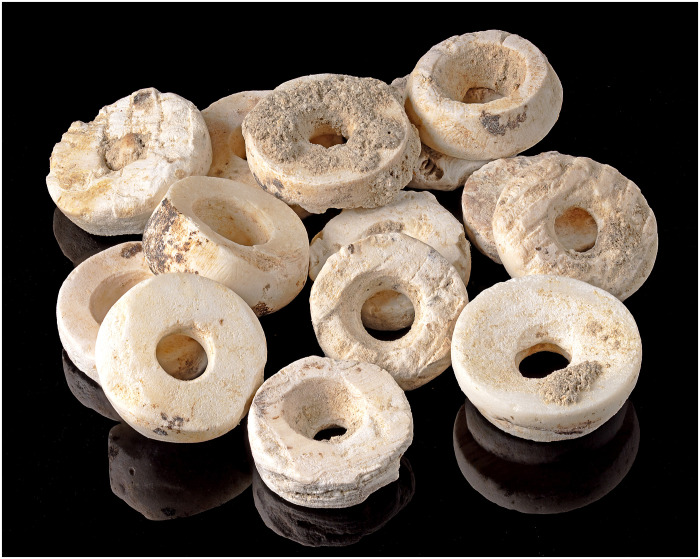
High-resolution photograph of beads from the upper level of Structure 10.049 (UE 535, above The Ivory Lady), attached to the rock crystal dagger. The photograph is a focus-stacked composite made following the same technique (and with the same equipment) described in Fig. 5. Photograph by D.W.W.

Next, samples were selected to estimate the total number of beads included in the assemblage and collect morphometric data (see below). Three samples were selected (see description in Materials and Methods). The first sample (Montelirio LC) produced weights between 0.19 and 0.01 g, with an average of 0.0708 g per bead, while the second (SC), with markedly smaller beads, yielded weights between 0.05 and 0.008 g, and an average of 0.0319 g per bead. On the basis on the total weight recorded divided by the average weight of individual beads, the estimated number of beads included in the assemblage is as follows: for the LC (7.71 kg), 108,898 beads; for the SC (3.42 kg), 107,210 beads. In total, 216,108 beads. This count, however, does not include the beads that were consolidated in situ and are kept separately in the museum, including especially individual UE-343. On the basis of an approximate weighting (excluding the wooden frame that holds the consolidate material and other elements), this material would amount to a further 3.87 kg of beads, that is to say, another 54,661 beads. Together, the entire Montelirio bead collection would amount to a remarkable 270,769 beads. This figure, which excludes the beads from Structure 10.042–10.049, probably sets the Montelirio bead assemblage as the largest ever recorded worldwide, as discussed below.

### Identification

**Fig. 7.**
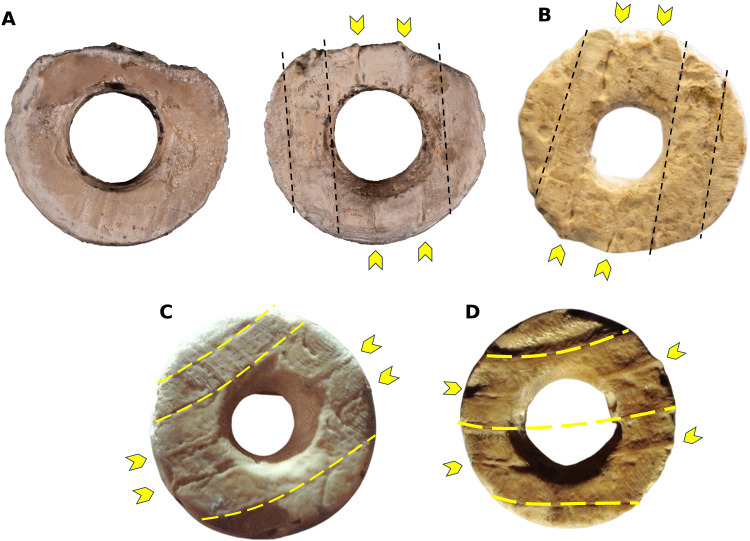
Details of the beads showing the different directions of the ribs. (**A**) Left: View of the inner side of the bead, right: view of the outer side showing the ornamentation. [(A) and **B**] Straight and subparallel ribs, corresponding to Pectinidae. (**C** and **D**) Slightly curved and parallel ribs, corresponding to Cardiidae. Design: F.M.G. and S.R.-C.

Basically, three types of beads were identified: those made of marine shell and a very small fraction made of stone and bone. The arrangement of the ribs detected on some of the beads, and their form, either straight or slightly curved, made it possible to identify at least two families of bivalve species (Fig. 7). The first family, or Pectinidae, includes beads with wide, straight and subparallel ribs. There are several species belonging to this family that can be found on the Atlantic coasts of the southern Iberia, including *Pecten maximus* and *Pecten jacobaeus* (*14*). The organisms belonging to this family live buried or semi-buried in the seabed, in shallow depressions, generally in places of fine, clean sand and sometimes in muddy areas, between rocks and crevices (*14*, *15*). These organisms live in conditions of water salinity of not less than 25‰ (*14*). They are found at depths that range from 10 to 183 m, being most common on the marine substrate at depths of 20 to 45 m (*14*).

As for the second family, Cardiidae, with narrow, slightly curved ribs, two possible genera were identified. Genus *Acanthocardia* includes five species, all of which occur in the Atlantic, namely, *Acanthocardia aculeata, Acanthocardia echinata, Acanthocardia paucicostata, Acanthocardia spinosa*, and *Acanthocardia tuberculata.* Species belonging to this genus can be found on well-sorted coarse or fine sandy, clayey sea beds, where they live buried (*16*, *17*). They are distributed in a range from 0 to 100 m depth (*17*, *18*), with the species *A. tuberculata* being very common at depths of 3 to 12 m on the Atlantic coasts of southern Iberia (*16*). On the other hand, two species of genus *Cerastoderma* were found, *Cerastoderma edule* and *Cerastoderma glaucum*, both of which can be found in the south Atlantic of Iberia (extracted from www.sealifebase.ca/). Individuals belonging to *C. edule* live buried at the bottom of shallow coastal areas and estuaries, in intertidal and subtidal zones composed of coarse-fine sand, silts, and sandy silts (*19*). On the other hand, *C. glaucum* lives on the bottom of lagoons and mudflats in calm waters, made up of poorly cohesive sediments (fine sand), where wave action does not reach (*19*). It is possible to establish a range of 0- to 50-m depth in which these species are distributed, being more common at levels closer to the coast and affected by tides (extracted from www.sealifebase.ca/summary/Cerastoderma-edule.html; www.marlin.ac.uk/species/detail/1315). The species *C. glaucum* can tolerate higher salinity levels as it lives in environments with higher salt concentration than *C. edule*, being respectively 15 to 35‰ and 12.5 to 38.5‰ (*19*).

Note that a small proportion of beads do not present the ornamentations typical of Pectinidae and Cardiidae. In some beads, the pearly layer (endostracum) of bivalves, commonly called mother of pearl, was recognized. In these, it is not possible to appreciate the characteristic iridescence of mother of pearl (possibly due to weathering or use-wear), but it may point to the use of other bivalves such as those of the genus *Ostrea* and *Anomia*. In these genera, the iridescence of the inner layer is much more evident than in Pectinidae and Cardiidae.

**Fig. 8.**
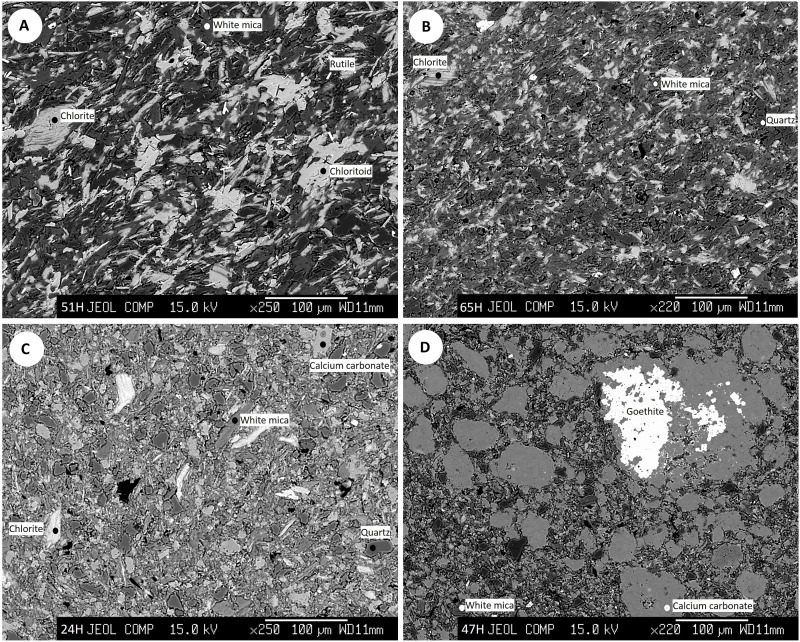
Scanning electron microscope images of lithic beads. (**A**) Chloritoid schist, (**B**) white mica-chlorite phyllite, (**C**) shale, and (**D**) limestone. Design: T.D.R.

As for the beads made of stone, in much smaller numbers, 10 beads of different colorations and grain sizes were selected. The following rock types, commonly displaying dark tones and colors ranging from gray to green (Fig. 8), were identified: chloritoid schists (samples 51H and 6G), white mica-chlorite phyllites (samples 65H and 5G), gray to green shales (samples 24H, 1G, 2G, and 3G), a meta-sandstone (sample 4G), and a green limestone (sample 47H). Chloritoid schists have a lepidoblastic texture and are composed of white mica + chlorite + chloritoid ± quartz ± rutile (Fig. 8A). The phyllites also show a lepidoblastic texture, and their main components are white mica + chlorite ± biotite ± albite ± quartz (Fig. 8B). Shales show a granolepidoblastic texture, and they are composed of white mica + quartz + chlorite ± calcite ± rutile (Fig. 8C). The green color of phyllites and shales is directly related to the chlorite content. The meta-sandstone has a less foliated fabric, a greater abundance of quartz than previous rocks, and smaller amounts of chlorite, white mica, and carbonates. Last, a greenish limestone with a detrital texture and some remains of fossil organisms, composed essentially of fragments of calcite and white mica, with quartz and goethite as accessory minerals (Fig. 8D), was observed.

Both the accessory nature of the lithic beads and the diversity of rocks used suggests that they were not extracted from a quarry but were collected opportunistically where accumulated and mixed with materials of rather diverse composition. Therefore, these rocks were detached from their original outcrop, eroded during transport, and deposited on riverbanks or coastal areas. All these rocks can be found at relatively short distances from the Valencina Copper Age mega-site. Chloritoid schists are rocks that, due to their mineralogy and the chemical composition of the chloritoid, can be related to equivalent rocks from the Cubito Formation. This geological formation is a highly deformed set of phyllites and schists outcropping north of the Aracena Massif (*20*). In addition to chloritoids, the chemical compositions of chlorites and white micas described in the schists and phyllites are also similar to those of the Cubito Formation (*21*). Gray to green shales, meta-sandstones, and limestones similar to those described have been observed around this geological formation [for example, in the Terena Formation, (*22*)], so the source area of all these materials could be located in the sector south of the Ossa-Morena Zone of the Iberian Massif (North-east of Aracena). These rocks could have been transported by the numerous streams, such as the Rivera de Huelva, that drain this area and are tributaries of the Guadalquivir River. All this evidence suggest that these beads were made with local material.

### Morphometry

The morphometric analysis of the Montelirio beads was mainly aimed at detecting differences in both their shape and/or size, looking at possible differences between the two chambers and their stratigraphic units (UE). The techniques used to achieve this are described in the Materials and Methods.

The results revealed differences in the size of the beads according to their location within Montelirio and Structure 10.042–10.049. This is evident in the average sizes, with the beads from the LC being noticeably larger and heavier than those recovered in Structure 10.042–10.049 and the SC of Montelirio (table S1). The nonparametric Kruskal-Wallis test confirmed the statistical significance of these differences in terms of all the measures considered during the analysis (table S2). However, the Bonferroni post hoc correction (which analyses one-to-one comparisons between pairs of sets) only partly qualified this conclusion, as statistical differences between the beads from Structure 10.042–10.049 and the Montelirio SC were not found in terms of both their maximum thickness and the maximum and minimum diameter of their perforations (table S3).

The principal components analysis based on the geometric morphometric analysis (GMA) data suggests that most beads are relatively similar to each other, with a comparatively smaller number of them showing a greater variability (figs. S1 and S2). This is the case even after the removal of c. 100 outliers from the sample (fig. S3, A to D). This apparent similarity seems to be supported by the discrete success of the linear discriminant analysis, which was only able to correctly classify 34.9% of the beads, although this success varies from 65.7% for structure 10.042 to only 2.8% for UE 209 (table S4).

However, other data derived from the GMA do indicate significant differences between the beads depending on the UE from which they originate. This is the case of the multivariate analysis of variance analysis (df: 5; H-L: 0.62296; approximately F: 5.1532; num. Df: 380; den. Df: 15717; Pr(>F): < 2.2 × 10^−16^) or the Haralick’s circularity. The latter is particularly interesting, as it evaluates how far the shape of an object is from being a perfect circle (the higher the value, the closer) (*23*). In the case of the Montelirio beads, circularity can be used as a proxy for the level of “perfection” or standardization achieved during their manufacturing process. The results (fig. S3E) show that the beads from Structure 10.049 and—especially—10.042 have not only an average circularity significantly higher than the rest of the UEs but also a considerably higher degree of internal variability (table S5). The Kruskal-Wallis analysis suggests that these differences in circularity are statistically significant (χ2 = 552.05, df = 5, *P* value < 2.2 × 10^−16^), although the Bonferroni correction makes it again necessary to qualify this statement (table S6), with no differences between—for example—the beads found in 10.049 and those recovered in UE 209 and 90.

### Chronometric modeling

A major goal of this study was to establish the chronology of the beaded attires. In light of the organic character of most beads, radiocarbon was deemed suitable. The specific aims were (i) to obtain an overall model of all beaded attires to establish their age range and a comparison of; (ii) the making of beads and the death of the group of people in the grave; (iii) the time of death of specific individuals and the making of the attires they wore when they were buried; (iv) the individual attires between them; (v) the use of Montelirio and the closely associated Structure 10.042–10.049 (grave of The Ivory Lady), particularly to test the hypothesis (*3*) that the beads found in the latter (alongside other grave goods) were deposited at the time of construction/use of Montelirio, sometime after the burial of The Ivory Lady. As explained in the Materials and Methods, 24 radiocarbon determinations were successfully obtained.

Four determinations were excluded from the Bayesian models: OxA-X-2535-32 (human bone), which dates an individual buried in the Montelirio corridor in the early centuries of the fifth millennium BC, long before Montelirio was built, Ua-40801 and Ua-40802 (human bone) from the Small Chamber, which was badly disturbed in Roman times, and OxA-32200, a date on charred material (unidentified roundwood twig) from the Montelirio main corridor, which yielded a much later age (Iron Age). To start with, note that the model based on human bone dates that showed the best overall agreement (Amodel: 95), suggested that the Montelirio tholos was first used (perhaps built) in 2875–2700 2σ cal BC and continued to be used until 2805–2635 2σ cal BC, being in use for a period of 1 to 200 years (95% probability) or 1 to 100 years (68% probability) (*2*).

First, a Bayesian model incorporating all 24 dates on shell beads revealed a start boundary in 3400–2995 2σ cal BC or 3250–3060 1σ cal BC and an end boundary set in 2595–2345 2σ cal BC or 2540–2420 1σ cal BC (fig. S4A), which are compatible with the model based on human bone. Of course, the large number of shells needed to manufacture the whole Montelirio bead assemblage must have involved a wide-ranging catchment along the neighboring coasts, probably involving shells of varying ages.

Next, Bayesian models were calculated for the beaded attires for which radiocarbon determinations were available (fig. S4B and table S8). For Individual UE102, an adult female aged 25 to 34 years at the time of death and wearing a full-body beaded tunic, two models were calculated. The first, one-phased model incorporated all dates available for this individual (three on bone and five on shell beads) and was not robust, with a verisimilitude index below 60%. Removing date OxA-41319, which is older, the model was still not robust. The one-phase model was only robust if both the oldest shell (OxA-41319) and bone (CNA-585) dates were removed. A second, two-phase model departs from the assumption that the shells for the beads were collected before the woman’s death and includes two sequences, one for the shell bead dates and another for the bone dates. This model was not sufficiently robust; however, if the oldest dates on shell (OxA-41319) and bone (CNA-585) were removed, then the resulting model is robust. Together, the first model suggests that it could reasonably be assumed that the shells were collected at or near the time of burial, while the second model suggests a collection of the shells beforehand. However, given the short time difference between both phases in the second model, as is shown in fig. S5A, the time difference between the collection of the shells, the making of the beads, and the death of the woman was quite short.

For individual UE103, a female aged 25 to 29 years, two models were also calculated. A one-phase model including all dates (one on shell and three on bone) was not statistically robust, as the shell bead date is discordant with the oldest date on bone. A second model assuming two phases also turned out not to be robust, even removing date OxA-28245, the oldest of the tree bone dates. Therefore, the bead appears to be posterior to the death of individual UE103.

Individual UE343, a female aged 24 to 32 years at the time of death, must have been a very special person. Not only she wore what seemingly was a full-body beaded tunic, but also she was also placed at a prominent location in the tomb, right in front of the unbaked-clay stela that presided over the center of the LC, on the path of the narrow projection of sunlight that came from outside on the summer solstice, and with both her arms raised above her shoulders and head, in a gesture frequently described as “oranti” in the literature on European late prehistory (*24*). Her bones yielded the lowest mercury levels in the Montelirio LC (*5*). A first, one-phase model incorporating all dates available for this woman (four on shell bead and one on bone) provided a statistically robust result. A second, two-phase model excluding the oldest shell bead date (fig. S5B) was also statistically robust. Consequently, similar to individual UE102, the shells were apparently collected shortly before or at the time of burial.

Regarding Structure 10.042–10.049, which basically is believed to have been originally built some two or three generations before Montelirio (*2*) and later revisited when Montelirio was built and/or in use, an interesting finding is that the two dates from the mother-of-pearl beads that decorated the rock crystal dagger deposited above The Ivory Lady are statistically identical. First, a single-phase model that included the three dates on human bone and two on shell bead from the first chamber (Structure 10.042) and the two dates on the beads of said dagger from the second chamber (Structure 10.049) turned out not to be statistically robust. Next, a second model was built excluding date CNA-1303 (on human bone) from the first chamber, older than the rest (which are basically coeval). This model turned out to be statistically robust (Amodel: 84); because they share a large proportion of the probability range, these determinations must date events occurring very closely in time.

Third, considering that the two dates of the rock crystal dagger beads are basically coeval with the individual dated by CNA-1291, a third, two-phase model was built, assuming a first phase in which the individual dated by CNA-1303, buried in Structure 10.042, was earlier than both the individual dated by CNA-1291 (in the same chamber) and the rock crystal dagger deposited in the upper level of second chamber (Structure 10.049). This model is very robust statistically (Amodel: 121), which suggest that the reuse of Structure 10.042–10.049 at the time of the construction and/or use of Montelirio involved both the deposition of high-end artifacts (such as the rock crystal dagger) and the deposition of human bodies (or bones). In this hypothesis, the temporal limits of Structure 10.042–10.049 would be as follows: First phase, start boundary set in 3150–2775 2σ cal BC or 2955–2880 1σ cal BC and end boundary set in 2925–2760 2σ cal BC or 2910–2855 1σ cal BC; second phase start boundary set in 2900–2710 2σ cal BC or 2885–2780 1σ cal BC and end boundary in 2870–2700 2σ cal BC or 2880–2610 1σ cal BC. This fully confirms the complex pattern of use/reuse between Structure 10.042–10.049 and the Montelirio tholos, which had been suggested in previous studies (*3*), and reveals the close cultural, social, and perhaps kin-based connection between the people buried in both tombs.

### Phytoliths

Two samples of soil solidified inside the perforations of two different beads were taken for phytolith and micro-archeological analysis. These beads came from the Montelirio LC and had not been submitted to in situ consolidation.

**Fig. 9.**
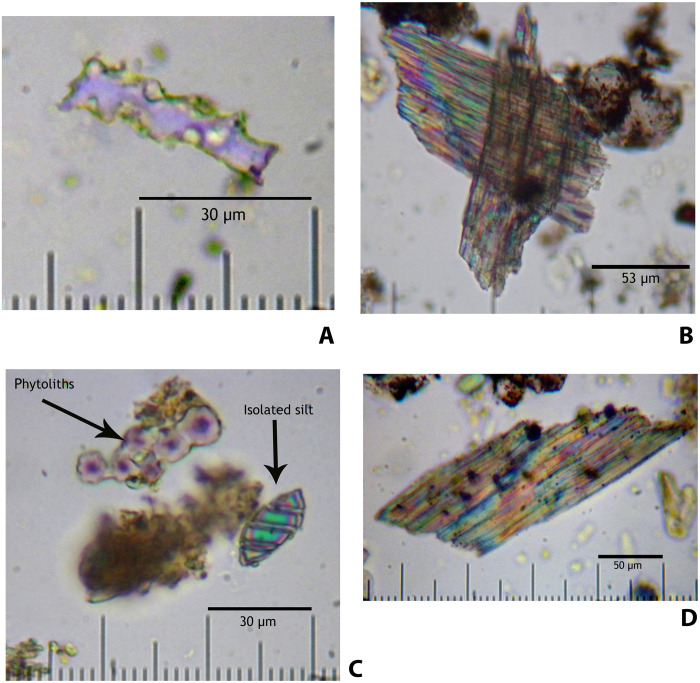
Micro-archaeological and phytolith analysis. (**A** and **B**) Sample “MONT-1 DJ-09 UE 355 N°B Ind. I”. (**C** and **D**) Sample “MONT-1 UE 94, N° B7”. Magnification: [(A) and (C)] ×600; [(B) and (D)] ×400. (A) Fragment of elongate phytolith, perhaps with echinate ornamentation in origin, proposed to be grasses (Poaceae) and belonging to the contextual sediment. (B) Microfragments of fibers proposed to be remains of *L. usitatissimum* (flax). (C) Phytolith of palmae (Arecaceae), here in apparent anatomical connection and probably belonging to *C. humilis*, with fusiform carbonated silt (23 μm × 18 μm) and clays micro-aggregates. (D) Microfragments of fibers proposed to be remains of *L. usitatissimum* (flax). Images: J.A.A.V.

Sample “MONT-1 DJ-09 UE 355 N°B Ind. I” (from the sediments surrounding the beads) yielded a single, small-sized phytolith fragment (<60 μm), probably of the long cell type associated with grasses (Poaceae). It was affected by siliceous dissolution (Fig. 9A). However, under the microscope, this sample revealed a series of microfiber fragments of birefringent optical behavior, in some cases superimposed, in what look like remains of a mesh, most probably connected with the fabric used to make the attires (Fig. 9B). These fragments are not siliceous, as they do show anisotropic bodies and an internal layered structure, similar to those present in certain plants with cellulose composition.

Sample “MONT-1 UE 94, N°B7” yielded several examples of globular/spherical phytoliths with echinate ornamentation that are typical, among others, of palmaceae (Arecaceae), probably belonging to *Chamaerops humilis* (shrub-like clumping palm), a species known in Iberia since at least the Miocene [(*25*) and Fig. 9C]. Similar phytoliths are also produced by palmaceae of the genus *Phoenix* (*26*), with known difficulty in identifying genera using only this type of phytoliths (*27*). Although little evidence exists for either in Iberian archeological contexts dating to late prehistory, *C. humilis* would appear as the most likely candidate, perhaps in connection with the fabric of the attires. Fragments of what appears to be a mesh of fibers forming a fabric were also found in this sample, with sizes always smaller than 200 μm in length and 70 μm in width (Fig. 9D). The shape and texture of these fiber fragments are not unlike those found in clothing made in *Linum usitatissimum* (flax) used in traditional rural Galicia (Spain), samples of which were observed under the microscope for this study. Flax is known to have been in use since the Upper Paleolithic (*28*) and is attested in Early Bronze Age Iberia (*29*, *30*).

### Experimental work

Experimental work on the production of shell beads was undertaken, following four steps, as described in Materials and Methods. Section S3 documents time invested in the various tasks preparing one bead for each shell species. Making one Cardiidae shell bead was more time-consuming (91 min on average) than making a Pectinidae one (19 min on average), as the former are thicker and have more marked ribs than the latter. Using the average of the time needed to make both types of beads (55 min), and the quantification of the whole assemblage described above, 270,000 beads, it would have taken 247,500 hours to make all beads found in Montelirio. However, it must be assumed that the crafts people who made the beads in the Copper Age were far more skilled at the job than modern archeologists. Therefore, if the average time is divided by five, then the resulting average time to make one bead would be 11 min, which would result in 49,500 hours for the production of the whole assemblage.

Next, an estimation was made of the volume of shells required to make all the beads. On the basis of the area of an average preform (as shown in fig. S6A2), which is 7.26 cm^2^, and considering the average area of a *P. maximus* shell (109.71 cm^2^), it was calculated that each shell would provide a maximum of 15 preforms. Assuming each preform yielded only one bead (which is reasonable, based on our experimental work), the total number of shells needed to produce the whole assemblage (in the lower range of the estimation, that is to say, 270,000 beads) would be 18,000. This estimate errs on the side of caution, as shells of Cardiidae are smaller and therefore a higher number of them would have been needed. Since, on average, a *P. maximus* shell valve weighs 44.6 g, this would represent 802 kg of marine shell involved in the production of the entire assemblage of beads.

In summary, 10 persons working 8 hours a day would have taken 206 days (nearly 7 months) to produce the whole assemblage using in the process a little less than a metric ton of marine shell. Evidently, the labor value of the beaded attires was high.

## DISCUSSION

The multidisciplinary approach deployed for this study has produced evidence for interpreting the remarkable Montelirio bead assemblage. Beads are a widespread and pervasive element of material culture produced by *Homo sapiens*. Excellent indicators of technology, social organisation, exchange patterns, and even beliefs, beads are a topic of research in their own right (*31*). Accordingly, the archeological and anthropological literature on these elements is quite vast. An annotated bibliography produced by the “Society of Bead Researchers” in 2016 and then updated in 2024 lists over 400 pages of references on this topic (*32*, *33*).

Examples of the extensive bead production and use abound since the Upper Paleolithic in both the New and Old Worlds. However, in quantitative terms, it is difficult to find a single-burial collection of discoidal beads of Montelirio’s magnitude, worldwide. At the site of Sunghir, in Russia, dating to c. 32,000 BC, grave 1 contained the remains of an adult male almost completely covered in c. 3000 mammoth ivory beads (as well as other personal ornaments), which are believed to have been sewn onto clothing (*34*). At Mal’ta, in Siberia, a child burial dating to between 23,000 and 19,000 uncal years before present was found in connection with a rich necklace of 120 bone beads associated with fragments of a tiara made from mammoth ivory, a large oval-shaped pendant, a round bone plaque with a zigzag pattern, and a figurine of a flying bird (*35*). At the Neolithic village of Ba′ja (7400–6800 BC), in Jordan, an 8-year-old child was found accompanied with more than 2500 beads that formed a complex ornament placed around its neck and chest (*36*). In Iberia, discoidal perforated beads, often made on marine shell, such as *P. maximus*, are documented since the Early Neolithic (c. 5400 BC) (*37*) and appear commonly in burials of the Copper Age (c. 3200–2300 BC) (*38*) and Early Bronze Age (c. 2300–1550 BC) (*39*). Two thousand years ago, the Chumash people of the Santa Cruz Island (California) produced millions of shell beads that were used as currency and circulated over hundreds of miles across North America, as far as Oregon, the Great Basin, and the Southwest (*40**–**42*).

Among prestate societies, published Paleolithic and Neolithic examples, such as those of Sunghir, Mal’ta, and Ba′ja involved several hundred or, at most, a few thousand beads. In North America, where bead making was substantial among the Chumash of California and in Cahokia, in the Mississippi region, during the first millennium AD, the best-known examples, including the “Beaded Burial” (or Mound 72) at Cahokia (containing the remains of a man and a woman), reach up to c. 30,000 beads at most (*41*, *43*, *44*). Of course, beads were widely used among early state societies of Mesopotamia and Egypt, in some cases involving spectacular attires, such as that of Queen Puabi of Ur’s (Mesopotamia) burial dress (or shroud), dated to c. 2500–2600 BC (*45*). However, in most cases, these attires were made of fewer tubular beads of precious stones such a lapis lazuli, carnelian, or agate and not mass-produced discoidal beads such as those found in Montelirio.

The calculations presented here suggest that the whole Montelirio bead assemblage could have been made in around 7 months by 10 persons working fulltime 8 hours a day. Obviously, any increase in the amount of labor deployed in its production would have reduced that time. Then, it does not appear as if the communities living at or around Valencina in the early centuries of the third millennium cal BC suffered from a shortage of labor. Together, the tasks involved in the processing of almost one metric ton of shells, which obviously had to be found on the nearby shores, collected, transported, and stored, added to the amount of work involved in the manufacturing of the beads.

The detailed chronometric models presented here suggest that the beads were manufactured near the time of death of the women who wore them. This raises the question of the temporality of use of Montelirio. The best-fitting chronometric model, based on human bone, does not exclude a relatively long period of use over a few decades (*2*). In that scenario, the beads might have reasonably been produced during that period of time. However, the radiocarbon model also does not exclude the possibility of a single burial event, with the women all dying at the same time, in which case the implications for the production would have been different. In that scenario, the attires may have been produced over a much shorter period of time. The chronology of the beads themselves supports both possibilities: They do not appear to have been “old” (for example, heirlooms) by the time they were deposited in the grave. However, at the same time, one of the dated beads is later than the person next to whom it was found, which runs counter to the “single burial event” hypothesis.

Either of those two scenarios is consistent with the growing evidence suggesting a rather “explosive” surge of early social complexity at Valencina in the period c. 2900–2650 BC. This is reflected in the deployment of a broad range of highly specialized crafts, involving sumptuous artifacts made of gold, ivory, rock crystal, amber, flint, mylonite, and marine shell, by the local elites. In light of the findings described above, sumptuary textiles must have played a part in the display and exhibition of authority and power and as part of the paraphernalia associated to the emerging elites. All dress is an expression of identity (*46*). For those sharing common cultural codes, attires can visually (and rapidly) inform about gender, age, and social standing, thus classifying people into groups. Specifically, dress is a crucial element in the performativity of gender (*47*, *48*). The social value of dresses may have been even higher in early complex prestate societies, with acquired, and not ascribed, social statuses.

In that context, weaving appears as an expanding technological and economic activity in Copper Age Iberia (*49*, *50*). Thus, the Montelirio beaded attires may well have been expressions of the collective identity of the people (mostly women) buried in it, including age, gender, and social standing (*46*). Therefore, the Montelirio beaded attires must be seen as another materialization of the process of identity construction through idiosyncratic crafts already noted for ivory artifacts (*6*), specially because some of the attires were further embellished with pendants made in ivory and amber, representing acorns, a bird, and other unidentified elements (*51*, *52*). Although Valencina offers many other examples of remarkable monumentality and refined craftmanship, the beaded attires may well have been restricted to the “peak” period occurring c. 2900–2650 BC at the site, as no other examples of large bead assemblages have been found to this date, the only other discoidal known beads being the very small collections found at La Pastora and the Depósito del Agua tholoi (which was completely destroyed in the 1980s).

While the evidence for textiles in the fourth and third millennia in Europe is elusive, there are examples of the use of clothing as a means to underline social ranking (*53*) and for ritual practice (*54*) dating to the Bronze Age. It is worth noting that of the Montelirio individuals wearing beaded garments, the two wearing the most complex attires (individuals UE102 and UE343, both female) (Fig. 2) were also those with the “upper-most” age at death (24 to 32 and 25 to 34 years of age, respectively). While it would be tempting to see some kind of “seniority” in this, clearly, this would need further support, as there is no probability involved in these age-of-death ranges. However, it is worth noting that their bodies were laid closely together in front of the clay “stela” (or altar) that dominated the scene and the closest to the access to the main corridor, which perhaps suggests that they were the last two people to be interred (*24*). Individual UE343 was placed in a supine position with her arms stretched above the head, a very unusual posture for an inhumed body in the Copper Age, while Individual UE102 was laid to rest on a prone position. Perhaps, this was the expression of different individual identities within what already was a highly distinctive, and most likely, powerful, social group. Differences in the attires (length, breadth, decoration, etc.) may have represented different gender categories and/or social rank, as testified by other known cases (*55*). At any rate, the high social standing (and, by inference, authority or power) of the women buried in Montelirio is underlined by the high quality of the material culture that was “sacrificed” (i.e., withdrawn from their social context) as part of their funeral, as is known to be often the case of textiles (*56*), which thus become a source of political power (*57*). In life, the Montelirio attires must have been used in very special occasions, for ceremonial and ritual purposes, in which they were probably seen by many people.

**Fig. 10.**
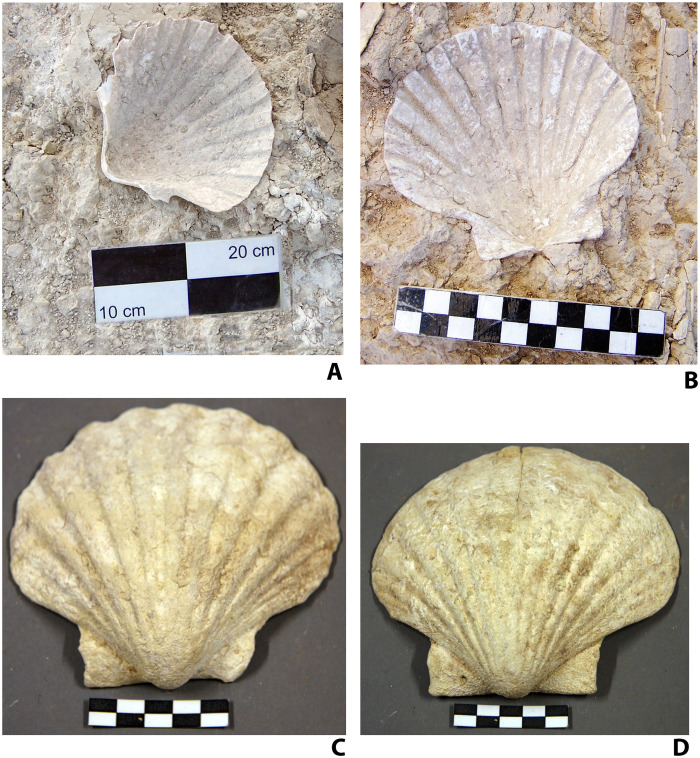
Examples of *P. maximus* valves found in graves and votive pits surrounding The Ivory Lady grave. (**A**) Structure 10.036 (negative feature with stone elements). (**B**) Structure 10.038 (negative feature without stone elements. (**C**) Structure 10.016 (pit burial). (**D**) Structure 10.022 (negative feature with stone elements). Photographs: (A and B) José Peinado Cucarella and (C and D) C. Mora Molina.

Another relevant question is why were shell beads, rather than other raw materials, chosen to emphasize the distinctiveness of the attires. Various lines of analysis must be considered. Most directly, the choice of shell beads was plausibly symbolic, associating the individuals with the sea. Up to 99% of the beads were made of seashell, obviously connecting to the marine environment surrounding Valencina 5000 years ago. The sea appears prominently in the worldview of the communities that inhabited and/or frequented Valencina in the first half of the third millennium BC. Sedimentary marine rocks, naturally “decorated” with erosive and biogenic motifs, were used for capstones and floor slabs of major megalithic monuments such as La Pastora or Matarrubilla (*58*, *59*), and a sperm-whale tooth was recently found in a nonburial pit at the Nueva Biblioteca sector. Marine symbolism is also reflected in the numerous valves of *P. maximus* used as grave goods and offerings in several of the burials and votive pits that were made around The Ivory Lady burial in the 200 or 250 years following her death (Fig. 10) (*60*).

The widespread use of *P. maximus* (scallop shell) as a highly symbolically charged material is connected with the use of other marine shells in Neolithic and Copper Age Europe, as is the case of *Spondylus* (*61*, *62*). This would appear to be relevant also in terms of the association of the beaded attires with women, both in Montelirio the LC and in The Ivory Lady grave. In Ancient Greece and Rome, of course, scallop shells were associated to the cult of Aphrodite/Venus (*63*). In turn, in Medieval and Modern Christianity, it was the symbol of Saint James and the Camino de Santiago (*64*).

Also, the symbolic use of color in the Montelirio attires may have been important. The marine shells, of “natural” and “marine” color, emphasize a sense of the “local” as proposed by Jones (*65*). Given the raw material chosen, the attires were white. That would have been the case, particularly, for the beads still featuring a nacreous or pearly layer. Under the sun, the effect of these women with the attires glittering with reflected sunlight must have been quite powerful, not the least because the contrasting red pigment from bright cinnabar powder was important in the ritual practice, perhaps even as body paint used by these people, as documented by the very high mercury levels in their bones (*5*). The presence of green and greenish beads combined with amber and ivory pendants would have increased the sensorial effect of the visually powerful dresses.

The choice of shell beads to distinguish elite burials may also be linked to the political economy of Valencina [sensu (*66*)]. Three linkages could be considered when studying this dimension of the Montelirio beads: (i) As discussed above, beads encapsulate high labor value representing the ability to use products representing substantial work. This work was specialized, measuring the ability to deploy (or to control) skilled labor. Could the intensive work involved in making beads mean that the attires were a “wearable” monument comparable in labor expenditure to traditions of megalithic monumentality but in a new medium but much harder to emulate by competitors? (ii) Second, requiring highly trained specialists, production of shell beads potentially may have involved some kind of patronage by elites able to mobilize resources in their support, and thus, it is possible to see how wealth availability could have been used and/or controlled by a new social group. Could the new use of multiple wealth items to distinguish special personages in Valencina document elite control over the making and use of special items of symbolic significance? (iii) Third, the use of shell beads, especially of fairly standardized forms as seen at Montelirio, is consistent with other evidence, suggesting the use of beads as an exchange medium across world prehistory and history (*41*). These shell beads probably fulfilled multiple functions, as Fauvelle describes, including objects of ceremonial use, marks of special identity, and exchange media associated with emerging maritime trade. The inventory of foreign, esoteric goods associated with these special burials suggests that the Montelirio burials required procuring special objects from afar [sensu (*67*)]. Could the use of shell beads in dress of special individuals represent a new source of economic power linked to established control (or perhaps monopolies) over maritime trade suggested by the coastal association of these monuments?

The morphometric analysis suggests the existence of differences between the assemblages recovered in the different chambers and stratigraphic units, both in terms of dimensions and shape. However, it is not clear whether these differences are merely due to the variability that should be expected from a non-industrial manufacturing process of hundreds of thousands of items or whether these differences were the result of different manufacturing actions, events, processes, or traditions. If the latter is the case, then this variation could be the result of the involvement of different groups of artisans (and/or workshops) over a relatively short period of time or, alternatively, could have been caused by changes in technical traditions, resulting from the lapse of one or several generations between the different episodes of manufacture/deposition of the huge set of beads recovered at the site.

In summary, the multimethod approach deployed in this study shows that being dressed with complex attires of thousands of beads was a special phenomenon in the Copper Age. Explaining the use of sophisticated textiles as both social markers and ritual paraphernalia in third millennium Iberia remains an open question that deserves further research. The same applies to the changing contexts, concentration, and significance of marine shell as isolated finds versus mass uses. That shell was important symbolically seems almost self-evident because of its distinctive uses in Montelirio and associated finds in the PP4-Montelirio and Nueva Biblioteca sectors as well as in the La Pastora and Matarrubilla tholoi. However, potential linkages to developing systems of distinction and ranking suggest important avenues for future investigation. Shell, along with other sources of wealth and symbolic significance, may be related to the idea of materialization of meaning. Identity (idiosyncrasies), distinction (social prominence), and special significance (worldview) blended in the Montelirio attires. These were important material elements establishing boundaries to how people could behave socially and individually. The choice of specific materials (the attires were largely made of shell and not stone, or bone, or ivory) conveys the physical forms of representation, which gave authority (and very probably, power) to some (versus use by many). The specifics of raw material procurement, manufacture, timing, use, and context suggest how prominent social practices and meaningful world views were created in third millennium BC Iberia.

## MATERIALS AND METHODS

To clean the beads, a three-stage process was followed. First, the beads were sieved with a fine mesh to eliminate small stones and soil; second, beads and the remaining soil were separated manually with wooden sticks and small brushes; third, an ultrasound machine was used to remove bits of soil attached to many beads, particularly inside perforations.

The photos presented in Figs. 5 and 6, intended to provide a detailed view of the morphology of the beads, are focus-stacked composites taken with a Canon R5 with MPE 65-mm f2.8 macro lens set at f5.6. The same ×3 magnification was maintained for every photograph. Lighting was achieved with two light-emitting diode (LED) panels, both camera and lights set to 5200 K to ensure consistent color representation. Camera was mounted on a computer-controlled, motorized macro rail (Cognisys Stackshot 3X), and raw format images were taken at 0.2-mm intervals resulting in a “stack” of 25 to 35 images for each bead. These were then combined using HeliconSoft “Helicon Focus” software, method B (depth map setting with radius = 8 and smoothing = 4; these parameters established by experimentation) to produce the resulting final images. Scale was established by photographing physical scales with identical settings and then adding the scale graphic to each image using Photoshop, at which time minor additional corrections of the background were also undertaken to remove dust, etc.

The 3D model of individual UE343 and her beaded attire, was created with 120 HD photographs and processed in Agisoft Metashape using the medium quality settings. The resulting 3D model has submillimetre accuracy. The control scale bars’ total error is 0.200 mm. The mesh has more than 713 k vertices.

To characterize the raw materials, the beads made in marine shell were observed and studied with a binocular loupe and a Levenhuk DTX TV digital lens. As for the lithic beads, because they have a variable grain size from very fine to microcrystalline, they were studied with a scanning electron microscope and an electron microprobe at the universities of Huelva and Granada to characterize them petrographically and determine their provenance.

For the morphometric analysis of the beads, three samples were selected. A first sample of 1738 beads (data S1) was selected, including at least 100 beads from each of the attires identified in the LC. A second sample (data S2) included 100 beads from the SC, which was badly destroyed in Roman times so that identification of individual attires was impossible. A third sample included 90 beads associated with the handle of the ivory and rock crystal dagger found in Structure 10.049, above the inhumation of The Ivory Lady (data S3). All beads were weighed using a high-precision scale (Fig. 4D). Two approaches were followed: a standard statistical analysis of various measurements and a GMA. For the first of the analyses, several measurements were taken (maximum diameter, maximum thickness and weight of the specimen, and maximum and minimum diameters of the perforation) from a sample of 1.738 complete items from both Structure 10.042–10.049 and Montelirio tholos (separating the SC and the LC). Meanwhile, the GMA was carried out over a sample of 3.360 complete beads recovered from both Structure 10.042–10.049 and the SC of Montelirio. To explore the differences between the beads recovered in the different stratigraphic units (or UEs) of Structure 10.042–10.049 and the Montelirio SC in greater detail, 3360 beads from both contexts were analyzed by means of GMA. This was performed using the elliptic fourier analysis method, which decomposes the outline of an object into a series of elliptical sinusoidal components (figs. S1 and S2). This method has already shown its potential for the analysis of archeological assemblages [e.g., (*68*, *69*)].

To establish the chronology of the beads, 24 determinations were obtained at the Oxford Radiocarbon Accelerator Unit, including two shell beads per attire, four in the case of full-body tunics, chosen from the random sample of 100 beads per attire selected for this study. These radiocarbon determinations were then statistically modeled in combination with the 24 already-published radiocarbon dates for Montelirio, 21 on human bone, and three on charred material, as well as the three dates on human bone available for Structure 10.042–10.049 (full data on all 48 dates are available in data S4; summary data of the 24 new dates in table S7) (*2*, *70*). For the statistical (Bayesian) modeling, all calibrated and modeled dates were rounded to 5 years, and the calculated phases and sequences are always sequential (i.e., it is assumed that a temporal lapse may exist between them). All dates on marine shell beads presented short SDs and were calibrated to a marine reservoir effect equivalent to −108 ± 31 14C year (*71*).

Phytolith analysis, following the methodology used in previous papers (*72*, *73*), was expected to shed some light on the fabrics used to weave the beads into complex elements of dress. Analyses were carried out both from the interior of the beads and the sediments around them. An inorganic dispersant (sodium hexametaphosphate) was used in the case of contextual sediments, but no reagents were used that could potentially eliminate any type of constituent elements from the samples. A reference collection available at the University of La Laguna was used for comparative purposes (*26*). Redundant tests with plants and sediments were carried out to ensure high-quality results.

Experimental work was undertaken to understand the technical skills and labor involved in production of the beads (*74*). Previous ethnographic and archeological studies were used as reference (*75*–*77*), and examples of Cardiidae and Pectinidae were gathered from the coastal areas near Sevilla along with raw materials necessary to replicate the tools available to the Copper Age craftspeople, including sandstone, quartzite, flint, pine resin, and wood. Four steps were involved: (i) extraction of the base-form by direct percussion on the shell with a lithic tool (fig. S6A); (ii) preliminary rounding of the base by abrasion (fig. S6, D4, F2, and F3); (iii) individual perforation of the bead through rotation of a flint-pointed drill (Fig. S6A, D and E); (iv) final rounding of the faces and edges. To make the perforations, a drill was created consisting of a flint point glued to a shaft with a compound made of pine resin and ash and then fastened with vegetal fibers (fig. S6, B1, B2, and B3). This drill worked by rubbing the shaft with both hands while holding the point firmly on the base-form; this process was repeated for both faces of the base-form to achieve a straight and conical perforation like those of the Montelirio beads (fig. S6C). Various versions of this drilling tools were made with points made of two different materials, quartz and flint, to test which was more efficient. As a result, flint points were deemed more efficient. The process described above is shown in fig. S6 (A, C, and D) for a shell of Pectinidae and in fig. S6 (E and F) for a shell of Cardiidae.

### Ethics statement

(i) The artifacts (discoidal shell beads) handled in this study were collected between 2007 and 2010 in the archaeological excavations undertaken at the Montelirio tholos under the direction of Á. Fernández Flores and at the PP4-Montelirio sector under the direction of J. Peinado Cucarella. (ii) The authenticity of the artifacts (discoidal shell beads) handled in this study was validated by Á. Fernández Flores and J. Peinado Cucarella, directors of the aforementioned excavations. The age/date validation and authentication of these artifacts were first made by the aforementioned directors of the excavations and were then confirmed by the radiocarbon dating presented here. (iii) All artifacts handled in this study are part of the permanent collection of the Sevilla Archaeology Museum and can be freely accessed for scientific purposes by authorization of the Museum director.

## Supplementary Materials

### The PDF file includes:

Sections S1 to S3 Tables S1 to S11 Figs. S1 to S6 Legends for data S1 to S4 References

## References

[1] A. Fernández Flores, L. García Sanjuán, M. Díaz-Zorita, Eds. *Montelirio: Un Gran Monumento Megalítico de la Edad del Cobre* (Junta de Andalucía, 2016). [Montelirio: A Great Copper Age Megalithic Monument].

[2] L. García Sanjuán, J. M. Vargas Jiménez, L. M. Cáceres Puro, M. E. Costa Caramé, M. Díaz-Guardamino Uribe, M. Díaz-Zorita Bonilla, A. Fernández Flores, V. Hurtado Pérez, P. M. López Aldana, E. Méndez Izquierdo, A. Pajuelo Pando, J. Rodríguez Vidal, D. W. Wheatley, A. Delgado-Huertas, E. Dunbar, A. Mora González, C. Bronk Ramsey, A. Bayliss, N. Beavan, D. Hamilton, A. Whittle, Assembling the dead, gathering the living: Radiocarbon dating and Bayesian modelling for Copper Age Valencina de la Concepción (Sevilla, Spain). J. World Prehist. 31, 179–313 (2018). 29962659 10.1007/s10963-018-9114-2 PMC5984651

[3] L. G. Sanjuán, M. L. Triviño, M. Cintas-Peña, Ivory, elites and lineages in Copper Age Iberia. Exploring the wider significance of the Montelirio tomb. Madrider Mitteilungen 59, 23–65 (2018).

[4] L. García Sanjuán, M. Cintas-Peña, M. Díaz-Guardamino, J. Escudero Carrillo, M. Luciañez Triviño, C. Mora Molina, S. Robles Carrasco, “Burial practices and social hierarchisation in Copper Age Southern Spain: Analysing tomb 10.042–10.049 of Valencina de la Concepción (Seville, Spain)” in *Megaliths, Societies, Landscapes. Early Monumentality and Social Differentiation in Neolithic Europe*, J. Müller, M. Hinz, M. Wunderlich, Eds. (Frühe Monumentalität und soziale Differenzierung 18/III, 2019), pp. 1005–1037.

[5] L. García Sanjuán, R. Montero Artús, S. Emslie, J. A. Lozano Rodríguez, M. Luciañez Triviño, Beautiful, magic, lethal: A social perspective of cinnabar use and mercury exposure at the Valencina Copper Age mega-site (Spain). J. Archaeol. Method Theory 31, 1006–1061 (2024).

[6] M. Luciañez Triviño, L. García Sanjuán, T. Schuhmacher, Crafting idiosyncrasies. Early social complexity, ivory and identity-making in Copper Age Iberia. Camb. Archaeol. J. 32, 23–60 (2021).

[7] M. Cintas-Peña, M. Luciañez-Triviño, R. Montero Artús, A. Bileck, P. Bortel, F. Kanz, K. Rebay-Salisbury, L. García Sanjuán, Amelogenin peptide analyses reveal female leadership in Copper Age Iberia (c. 2900-2650 BC). Sci. Rep. 13, 9594 (2023). 37414858 10.1038/s41598-023-36368-x PMC10326254

[8] L. García Sanjuán, C. Scarre, D. W. Wheatley, The mega-site of Valencina de la Concepción (Seville, Spain): Debating settlement form, monumentality and aggregation in southern Iberian Copper Age societies. J. World Prehist. 30, 239–257 (2017).

[9] J. Guilaine, Siret’s Smile. Antiquity 92, 1247–1259 (2018).

[10] A. Whittle, *Times of Their Lives. Hunting History in the Archaeology of Neolithic Europe* (Oxbow Books, 2017).

[11] B. Gaydarska, J. Chapman, *Megasites in Prehistoric Europe. Where Strangers and Kinsfolk Met* (Cambridge Univ. Press, 2022).

[12] M. Díaz-Guardamino, D. W. Wheatley, E. F. Williams, J. A. Garrido Cordero, “Los textiles elaborados con cuentas perforadas de Montelirio” in *Montelirio: Un Gran Monumento Megalítico de la Edad del Cobre*, Seville, A. Fernández Flores, L. García Sanjuán, M. Díaz-Zorita Bonilla, Eds. (Junta de Andalucía, 2016), chap. 14, pp. 345–365. [Montelirio’s textiles made with perforated beads].

[13] J. C. Pecero Espín, “Caracterización antropológica de los restos óseos humanos del tholos del Montelirio” in *Montelirio: Un Gran Monumento Megalítico de la Edad del Cobre, Sevilla*, A. Fernández Flores, L. García Sanjuán, M. Díaz-Zorita Bonilla, Eds. (Junta de Andalucía, 2016), chap. 16, pp. 409–442. [Anthropological characterization of human skeletal remains from the Montelirio tholos].

[14] A. R. Brand, “Scallop ecology: distributions and behaviour” in *Developments in aquaculture and fisheries science* (Elsevier, 2006), pp. 651–744, vol. 35.

[15] D. Minchin, Introductions: Some biological and ecological characteristics of scallops. Aqua. Living Res. 16, 521–532 (2003).

[16] M. M. Rufino, M. B. Gaspar, A. M. Pereira, F. Maynou, C. C. Monteiro, Ecology of megabenthic bivalve communities from sandy beaches on the south coast of Portugal. Sci. Mar. 74, 163–178 (2010).

[17] M. Peharda, D. Ezgeta-Balić, M. Radman, N. Sinkjević, N. Vrgoč, I. Isajlović, Age, growth and population structure of Acanthocardia tuberculata (Bivalvia: Cardiidae) in the eastern Adriatic Sea. Scientia Marina 76, 59–66 (2012).

[18] A. Rharrass, M. Talbaoui, M. Gaspar, M. Kabine, N. Rharbi, Gametogenic cycle of the rough cockle Acanthocardia tuberculata (Mollusca: Bivalvia) in the M’diq Bay (SW Mediterranean Sea). Sci. Mar. 80, 359–368 (2016).

[19] S. K. Malham, T. H. Hutchinson, M. Longshaw, A review of the biology of European cockles (Cerastoderma spp.). J. Mar. Biol. Assoc. United Kingdom 92, 1563–1577 (2012).

[20] J. P. Bard, “Le Metamorphisme Regional Progressif des Sierras d’Aracena en Andalousie Occidentale (Espagne). Sa Place dans le Segment Hercynien Sub-Iberique,” thesis, Montpellier (1969). [The Regional Progressive Metamorphism of the Sierras de Aracena in Western Andalusia (Spain). Its place in the Sub-Iberian Hercynian Segment].

[21] C. Ponce, J. F. Simancas, A. Azor, D. J. Martínez Poyatos, G. Booth-Rea, I. Expósito, Metamorphism and kinematics of the early deformation in the Variscan suture of SW Iberia. J. Metam. Geol. 30, 625–638 (2012).

[22] IGME (2015): *Mapa Geológico de España. Escala 1:200.000. Sevilla*–*Puebla de Guzmán* [Geological and Mining Institute of Spain (IGME), 2015]. [Geological Map of Spain. Scale 1:200.000. Sevilla-Puebla de Guzmán].

[23] R. M. Haralick, A measure for circularity of digital figures. IEEE Trans. Syst. Man Cybern. SMC-4, 394–396 (1974).

[24] L. García Sanjuán, A. Fernández Flores, M. Díaz-Zorita Bonilla, “Montelirio. Valoración e interpretación de una tumba excepcional” in *Montelirio: Un Gran Monumento Megalítico de la Edad del Cobre*, Seville, A. Fernández Flores, L. García Sanjuán, M. Díaz-Zorita Bonilla, Eds. (Junta de Andalucía, 2016), chap. 22, pp. 503–553. [Montelirio. Assessment and interpretation of an exceptional tomb].

[25] A. Pinilla, M. A. Bustillo, Silicofitolitos en secuencias arcillosas con silcretas. Mioceno Medio, Madrid. Monografías del Centro de Ciencias Medioambientales 4, 255–265 (1997). [Silicophytoliths in clay sequences with silcretes].

[26] J. A. Afonso Vargas, “Aplicación del Análisis de Fitolitos y otros Microfósiles al Estudio de Yacimientos, Materiales Arqueológicos y Edáficos de las Islas Canarias. Los Ejemplos de Las Cañadas del Teide (Tenerife), La Cerera (Arucas, Gran Canaria) y Otras Zonas de Aplicación Experimental”, thesis, University of La Laguna, Santa Cruz de Tenerife, Spain (2014). [Application of the Analysis of Phytoliths and other Microfossils to the Study of Archaeological and Edaphic Materials of the Canary Islands. The Examples of Las Cañadas del Teide (Tenerife), La Cerera (Arucas, Gran Canaria) and Other Areas of Experimental Application].

[27] D. Piperno, *Phytoliths, A Comprehensive Guide for Archaeologists and Paleoecologists* (AltaMira Press, 2006).

[28] E. Kvavadze, O. Bar-Yosef, A. Belfer-Cohen, E. Boaretto, N. Jakeli, Z. Matskevich, T. Meshveliani, 30,000-year-old wild flax fibers. Science 325, 1359–1359 (2009). 19745144 10.1126/science.1175404

[29] C. Alfaro-Giner, Spain in *Textiles and Textile Production in Europe from Prehistory to AD 400*, M. Gleba, U. Mannering, Eds. (Oxbow Books, 2012), pp. 334–346.

[30] F. Molina González, M. O. Rodríguez Ariza, S. Jiménez-Brobeil, M. Botella, La Sepultura 121 del yacimiento argárico de El Castellón Alto (Galera, Granada). Trabajos de Prehistoria 60, 153–158 (2003). [Burial 121 of the Argaric site of El Castellón Alto].

[31] D. Bar Yosef Mayer, C. Bonsall, A. M. Choyke, Eds., *Not Just for Show. The Archaeology of Beads, Beadwork and Personal Ornaments* (Oxbow Books, 2017).

[32] K. Karklins, *Researching The World’s Beads. An Annotated Bibliography* (Society of Bead Researchers, 2016).

[33] K. Karklins, *Researching The World’s Beads. An Annotated Bibliography*. *Archaeometric Analysis* (Society of Bead Researchers, 2024).

[34] E. Trinkaus, A. P. Buzhilova, Diversity and differential disposal of the dead at Sunghir. Antiquity 92, 7–21 (2018).

[35] L. Lbova, The Siberian Paleolithic site of Mal’ta: A unique source for the study of childhood archaeology. Evol. Hum. Sci. 3, e9 (2021). 37588521 10.1017/ehs.2021.5 PMC10427291

[36] H. Alarashi, M. Benz, J. Gresky, A. Burkhardt, A. Fischer, L. Gourichon, M. Gerlitzki, M. Manfred, J. Sakalauskaite, B. Demarchi, M. Mackie, M. Collins, C. Odriozola, J. A. Garrido Cordero, M. A. Avilés, L. Vigorelli, A. Re, H. G. K. Gebel, Threads of memory: Reviving the ornament of a dead child at the Neolithic village of Ba`ja (Jordan). PLOS ONE 18, e0288075 (2023). 37531349 10.1371/journal.pone.0288075 PMC10396020

[37] J. L. Pascual Benito, “Los talleres de cuentas de Cardium en el Neolítico peninsular” in *Actas III Congreso de Neolítico en la Península Ibérica (Santander, 5–8 octubre de 2003)*, P. Arias Cabal, R. Ontañón Peredo, C. García-Moncó Piñeiro, Eds. (Monografías del Instituto Nacional de Investigaciones Prehistóricas de Cantabria, 2005), pp. 227–286. [The bead workshops of Cardium in the peninsular Neolithic period].

[38] M. I. Dias, Z. Kasztovszky, M. I. Prudêncio, I. Harsányi, I. Kovács, Z. Szőkefalvi-Nagy, J. Mihály, G. Káli, A. C. Valera, A. L. Rodrigues, Investigating beads from Chalcolithic funerary cremation contexts of Perdigões, Portugal. J. Archaeol. Sci. 20, 434–442 (2018).

[39] M. Oliva Poveda, Els ornaments personals de la primera meitat del segon mil·lenni ane del jaciment de Can Roqueta-II (Est), Sabadell. Cypsela 15, 229–249 (2004). [Personal ornaments from the first half of the second millennium BCE from the site of Can Roqueta-II (east), Sabadell].

[40] M. Fauvelle, Mobile Mounds: Asymmetrical exchange and the role of the Tomol in the development of Chumash complexity. California Archaeol. 3, 141–158 (2011).

[41] M. Fauvelle, *Shell Money. A Comparative Study* (Cambridge Univ. Press, 2024).

[42] L. H. Gamble, The origin and use of shell bead money in California. J. Anthropol. Archaeol. 60, 101237 (2020).

[43] M. L. Fowler, J. Rose, B. Vander Leest, S. R. Aler, *The Mound 72 Area: Dedicated and Sacred Space in Early Cahokia* (Illinois State Museum Reports of Investigations no. 54, Santa Fe, Ancient City Press, 1997).

[44] L. Kozuch, Shell bead crafting at Greater Cahokia. North American Archaeologist 43, 64–94 (2022).

[45] A. Baadsgaard, All the Queen’s Clothes: Identifying Female Royalty at Early Dynastic Ur (Near Eastern Archaeology, 2016), vol. 79, pp. 148–155.

[46] P. Shukla, *Costume: Performing Identities through Dress* (Indiana Univ. Press, 2015).

[47] J. Butler, *Gender Trouble. Feminism and the Subversion of Identity* (Nueva York, 1999).

[48] E. Robertson Martinez, “Social Representations and Women Who Live as Men in Northern Albania” thesis, University of Cambridge, England (2020).

[49] M. Diniz, “Pesos de tear e tecelagem no Calcolitico em Portugal”, *Actas do 1er Congresso de Arqueologia Peninsular (Porto, 12*–*18 de Outubro de 1993)*. 4, 133–146 (1994). [Weights for looms and weaving in the Chalcolithic period in Portugal].

[50] L. M. C. Rollán, Las manufacturas textiles en la Prehistoria: Las placas de telar en el Calcolítico peninsular. Zephyrus 49, 125–145 (1996). [Textile Manufactures in Prehistory: The loom plates in the peninsular Chalcolithic period].

[51] M. Murillo-Barroso, “El ámbar del tholos de Montelirio” in *Montelirio: Un Gran Monumento Megalítico de la Edad del Cobre, Sevilla*, A. Fernández Flores, L. García Sanjuán, M. Díaz-Zorita Bonilla, Eds. (Junta de Andalucía, 2016), chap. 13, pp. 311–344. [Amber from the Montelirio tholos].

[52] M. Luciañez Triviño, L. García Sanjuán, “Los marfiles del tholos de Montelirio” in *Montelirio: Un Gran Monumento Megalítico de la Edad del Cobre, Sevilla*, A. Fernández Flores, L. García Sanjuán, M. Díaz-Zorita Bonilla, Eds. (Junta de Andalucía, 2016), chap. 10, pp. 245–272. [The ivories of the Montelirio tholos].

[53] K. Grömer, H. Rösel-Mautendorfer, L. B. Jørgensen, Visions of dress: Recreating Bronze Age clothing from the Danubian region. Textile J. Cloth Cult. 11, 218–241 (2013).

[54] J. Gulizio, Textiles for the Gods? Linear B evidence for the use of textiles in religious ceremonies in *Kosmos: Jewellery, Adornment and Textiles in the Aegean Bronze Age. Proceedings of the 13th International Aegean Conference/13e Rencontre Égéenne Internationale, University of Copenhagen, Danish National Research Foundation’s Centre for Textile Research, 21–26 April 2010*, M.-L. Nosch, R. Laffineur, Eds. (Aegaeum 33 series, 2012), pp. 279–285.

[55] M. L. Sørensen, Reading dress: The construction of social categories and identities in Bronze Age Europe. J. European Archaeol. 5, 93–114 (1997).

[56] J. Schneider, The anthropology of cloth. Ann. Rev. Anthropol. 16, 409–448 (1987).

[57] J. Schneider, A. B. Weiner, Cloth and the organization of human experience. Curr. Anthropol. 27, 178–184 (1986).

[58] L. M. Cáceres-Puro, F. Muñiz Guinea, J. Rodríguez Vidal, J. M. Vargas, T. Donaire, Marine bioerosion in rocks of the prehistoric *tholos of La Pastora* (Valencina de la Concepción, Seville, Spain): Archaeological and palaeoenvironmental implications. J. Archaeol. Sci. 41, 435–446 (2014).

[59] L. M. Cáceres, J. M. Vargas, F. Muñiz, T. Donaire, L. García Sanjuán, C. Odriozola, J. Rodríguez-Vidal, Natural “megalithic art” at Valencina (Seville): A geoarchaeological approach to stone, architecture, and cultural choice in Copper Age Iberia. Archaeol. Anthropol. Sci. 11, 4621–4641 (2019).

[60] C. L. von Lettow-Vorbeck, M. T. Aparicio Alonso, R. Araujo, L. Llorente-Rodriguez, A. Morales-Muñiz, La fauna del Sector PP4-Montelirio del yacimiento prehistórico de Valencina de la Concepción (Sevilla). Economía y simbolismo de los animales en una comunidad del III milenio. Menga Revista de Prehistoria de Andalucía 4, 69–102 (2014). [The fauna of the PP4-Montelirio Sector of the prehistoric site of Valencina de la Concepción].

[61] J. Chapman, B. Gaydarska, V. Slavchev, “The life histories of Spondylus shell rings from the Varna I Eneolithic cemetery (north-east Bulgaria): transformation, revelation, fragmentation and deposition”, in VVAA: *The Varna Eneolithic Necropolis and Problems of Prehistory in Southeast Europe*, V. Slavchev, Ed. (Acta Musei Varnaensis, 2008), pp. 139–162.

[62] F. Ifantidis, M. Nikolaidou, Eds., *Spondylus in Prehistory: New Data and Approaches. Contributions to the Archaeology of Shell Technologies* (British Archaeological Reports, International Series 2216, Archaeopress, 2011).

[63] J. Laver, “The Cradle of Venus” in *The Scallop: Studies of a Shell and its Influences on Humankind*, I. Cox, Ed. (Shell, 1957), pp. 73–89.

[64] C. Hobler, “The Badge of St James” in *The Scallop: Studies of a Shell and its Influences on Humankind*, I. Cox, Ed. (Shell, 1957), pp. 49–72.

[65] A. Jones, Local colour: Megalithic architecture and colour symbolism in Neolithic Arran. Oxf. J. Archaeol. 18, 339–350 (1999).

[66] T. Earle, M. Spriggs, Political economy in prehistory: A Marxist approach to pacific sequences. Curr. Anthropol. 56, 515–544 (2015).

[67] M. Helms, *Ancient Panama: Chiefs in Search of Power* (Texas Press, 1976).

[68] C. S. Hoggard, J. McNabb, J. Cole, The application of elliptic Fourier analysis in understanding biface shape and symmetry through the British Acheulean. J. Paleo. Arch. 2, 115–133 (2019).

[69] L. Timbrell, P. de la Peña, A. Way, C. Hoggard, L. Backwell, F. d’Errico, L. Wadley, M. Grove, Technological and geometric morphometric analysis of ‘post-Howiesons Poort points’ from Border Cave, KwaZulu-Natal, South Africa. Quaternary Sci. Rev, 297, 107813 (2022).

[70] A. Bayliss, N. Beavan, C. Bronk Ramsey, A. Delgado-Huertas, M. Díaz-Zorita Bonilla, E. Dunbar, “La cronología radiocarbónica del tholos de Montelirio” in *Montelirio: Un Gran Monumento Megalítico de la Edad del Cobre, Seville*, A. Fernández Flores, L. García Sanjuán, M. Díaz-Zorita Bonilla, Eds. (Junta de Andalucía, 2016), chap.21, pp. 485–502. [The radiocarbon chronology of the Montelirio tholos].

[71] J. M. M. Martins, A. M. M. Soares, Marine radiocarbon reservoir effect in Southern Atlantic Iberian coast. Radiocarbon 55, 1123–1134 (2013).

[72] C. R. Arbelo Rodríguez, J. A. Afonso Vargas, A. Rodríguez Rodríguez, Estudio preliminar de fitolitos en suelos agrícolas de Tenerife (Islas Canarias). Cereales y otros grupos vegetales in *Retos y Oportunidades en la Ciencia del Suelo. Actas del VI Congreso Ibérico de la Ciencia del Suelo (Santiago de Compostela 22–25 de junio de 2014)*, F. Macías Vázquez, M. Díaz Raviña, M. T. Barral Silva, Eds. (Andavira, 2014), pp. 37–40. [Preliminary study of phytoliths in agricultural soils of Tenerife (Canary Islands). Cereals and other plant groups].

[73] J. Afonso-Vargas, I. La Serna-Ramos, M. Arnay-de-la-Rosa, Fungal spores located in 18th century human dental calculi in the church “La Concepción” (Tenerife, Canary Islands). J. Archaeol. Sci. Rep. 2, 106–113 (2015).

[74] S. Ramírez-Cruzado, “Las Cuentas Perforadas del Tholos de Montelirio (Castilleja de Guzmán, Sevilla). Una Aproximación Comparativa, Geoarqueológica y Experimental,” thesis, University of Sevilla, Sevilla, Spain (2020). [The Perforated Beads from the Montelirio tholos (Castilleja de Guzmán, Sevilla). A Comparative, Geoarchaeological and Experimental Approach].

[75] B. Malinowski, *Argonauts of the Western Pacific: An Account of Native Enterprise and Adventure in the Archipelagoes of Melanesian New Guinea* (Routledge, 1922).

[76] S. Leonardt, “Artifactos Malacológicos en el Bosque y Ecotono Bosque-Estepa del Noroeste de Patagonia,” thesis University of Buenos Aires, Argentina (2013). [Malacological Artifacts in the Forest and Forest-Steppe Ecotone of Northwest Patagonia].

[77] S. Leonardt, La elaboración de cuentas con valvas de moluscos en Patagonia a través de la arqueología experimental. Comechingonia 23, 279–302 (2019). [The elaboration of beads with mollusk shells in Patagonia through experimental archaeology].

[78] C. Perlès, P. Pion, The Cerastoderma bead production at Franchthi (Greece): A case of apprenticeship? in *Beauty and the Eye of the Beholder: Personal Adornments Across the Millenia*., M. Mărgărit, A. E. Boroneanţ, Eds. (Editura Cetatea de Scaun, 2020).

[79] M. Guinea, Un sistema de producción artesanal de cuentas de concha en un contexto doméstico manteño: Japoto (provincia de Manabí, Ecuador). Bulletin de l’Institut français d’études andines 35, 299–312 (2006). [An artisanal production system of shell beads in a manteño domestic context: Japoto].

