# Supplementary material for: A multi-analytical study of the Montelirio beaded attires: Marine resources, sumptuary crafts, and female power in copper age Iberia
Source: Sci Adv. 2025 Jan 29;11(5):eadp1917. doi: 10.1126/sciadv.adp1917 (PMC11777232; doi:10.1126/sciadv.adp1917)
Supplement: Supplementary file 1 — Sections S1 to S3 Tables S1 to S11 Figs. S1 to S6 Legends for data S1 to S4 References [file sciadv.adp1917_sm.pdf]

Science Advances
AAAS

Supplementary Materials for

A multi-analytical study of the Montelirio beaded attires: Marine resources, sumptuary crafts and female power in copper age Iberia

Leonardo García Sanjuán *et al.*

Corresponding author: Samuel Ramírez-Cruzado, samramagu@alum.us.es

Sci. Adv. **11**, eadp1917 (2025)
DOI: 10.1126/sciadv.adp1917

The PDF file includes:

Sections S1 to S3
Tables S1 to S11
Figs. S1 to S6
Legends for data S1 to S4
References

Other Supplementary Material for this manuscript includes the following:

Data S1 to S4

SUPPLEMENTARY MATERIAL 1. MORPHOMETRIC DATA

PART 1. STATISTICAL ANALYSIS OF THE MEASUREMENTS OBTAINED ON A SAMPLE OF 1.738 BEADS FROM MONTELIRIO.

VARIABLE		CHAMBER		
		10042-10049	LC	SC
Maximum Diameter (mm.)	Mean	4.44	5.33	3.90
	Minimum	2.90	3.00	2.00
	Maximum	6.80	7.00	5.50
	Median	4.50	5.20	4.00
Maximum Thickness (mm.)	Mean	1.50	1.66	1.58
	Minimum	0.70	0.60	0.90
	Maximum	3.00	3.80	2.30
	Median	1.50	1.80	1.60
Weight (gr.)	Mean	0.05	0.07	0.03
	Minimum	0.01	0.01	0.01
	Maximum	0.18	0.20	0.06
	Median	0.05	0.07	0.03
Max. Diam. Perforation (mm.)	Mean	2.16	2.53	2.04
	Minimum	1.00	1.00	1.00
	Maximum	3.50	6.00	4.10
	Median	2.00	2.50	2.00
Min. Diam. Perforation (mm.)	Mean	1.92	2.04	1.74
	Minimum	1.00	0.80	1.00
	Maximum	3.00	4.50	2.90
	Median	2.00	2.00	1.75

Table S1. Descriptive statistics of the bead measurements according to the sector of Montelirio in which they were found.

Variable	Kruskal-Wallis chi-squared	df	p-value
**Maximum Diameter**	387.39	2	< 2.2e-16*
**Maximum Thickness**	23.232	2	9.021e-06*
**Weight**	293.01	2	< 2.2e-16*
**Max. Diam. Perforation**	110.6	2	< 2.2e-16*
**Max. Diam. Perforation**	47.168	2	5.723e-11*

Table S2. Kruskal-Wallis test of bead measurements according to the sector of Montelirio in which they were found (* significant at 0.05).

Variable	Sector	Z	P.unadj	P.adj
**Maximum Diameter**	10042-10049 vs. LC	-13.837752	1.51E-43	4.525542e-43*
	10042-10049 vs. SC	3.672675	2.40E-04	7.200750e-04*
	LC vs. SC	15.099406	1.63E-51	4.901995e-51*
**Maximum Thickness**	10042-10049 vs. LC	-4.3994076	1.09E-05	3.256404e-05*
	10042-10049 vs. SC	-0.8751255	3.82E-01	1.00E+00
	LC vs. SC	2.3282482	1.99E-02	5.97E-02
**Weight**	10042-10049 vs. LC	-10.98346	4.59E-28	1.377018e-27*
	10042-10049 vs. SC	4.57816	4.69E-06	1.407253e-05*
	LC vs. SC	13.99758	1.61E-44	4.838173e-44*
**Max. Diam. Perforation**	10042-10049 vs. LC	-7.870474	3.53E-15	1.059897e-14*
	10042-10049 vs. SC	1.277505	2.01E-01	6.04E-01
	LC vs. SC	7.606068	2.83E-14	8.476841e-14*
**Min. Diam. Perforation**	10042-10049 vs. LC	-3.736602	1.87E-04	5.595714e-04*
	10042-10049 vs. SC	2.624499	8.68E-03	2.60E-02
	LC vs. SC	6.053352	1.42E-09	4.255865e-09*

Table S3. Bonferroni post-hoc test of bead measurements according to the sector of Montelirio in which they were found (* significant at 0.05).

PART 2. GEOMETRIC MORFOMETRIC ANALYSIS USING THE ELLIPTIC FOURIER ANALYSIS METHOD OVER A SAMPLE OF 3.360 BEADS RECOVERED FROM DIFFERENT STATIGRAPHIC UNITS OF 10.042-10.049 AND THE SC OF MONTELIRIO.

These analyses were performed in R version 4.1.1. and the package Momocs version 1.4.0. The code used for this calculations was partially adapted from Timbrell et al. (70).

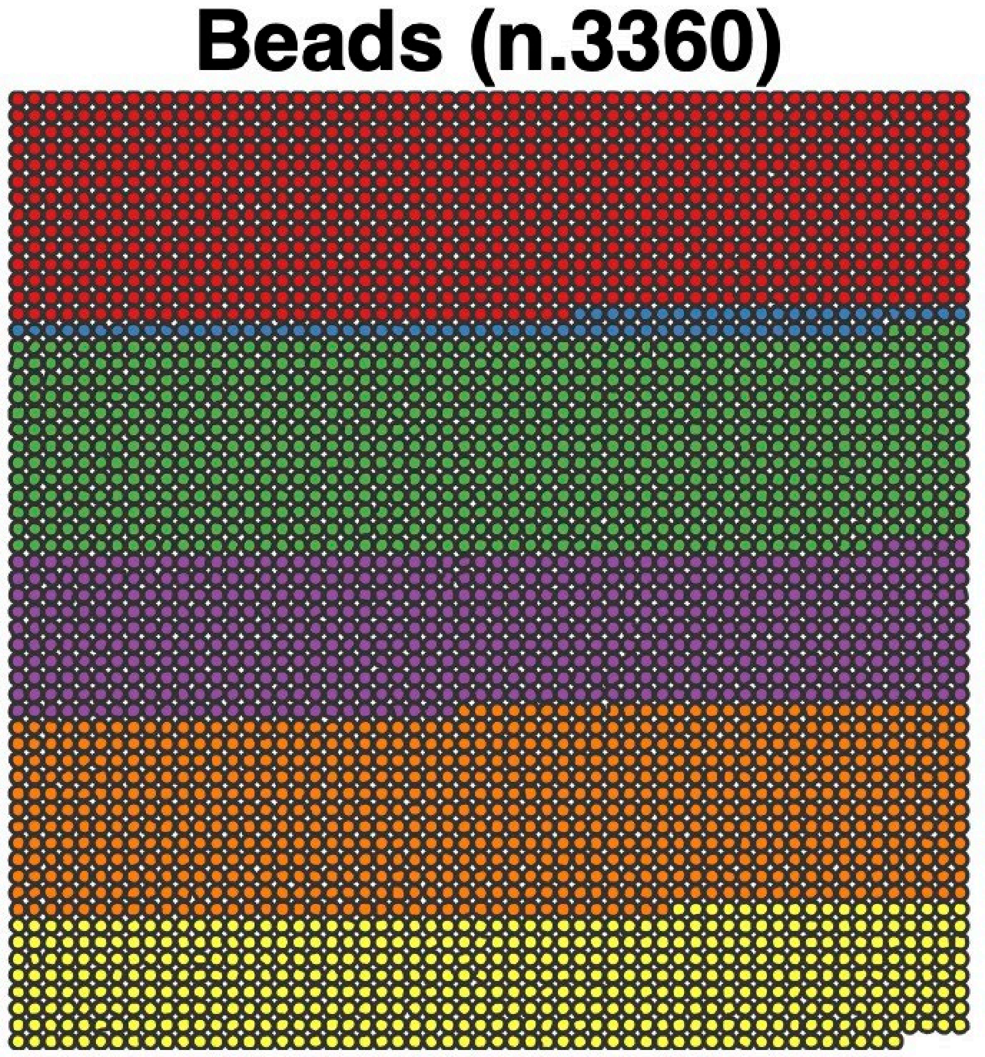

Figure S1. Outlines of the 3360 beads from Montelirio used for the GMA analysis. Red: 10.042; Blue: 10.049; Green: UE 80; Purple: UE 88; Orange: UE 90; Yellow: UE 209. Design: Carlos Rodríguez Rellán.

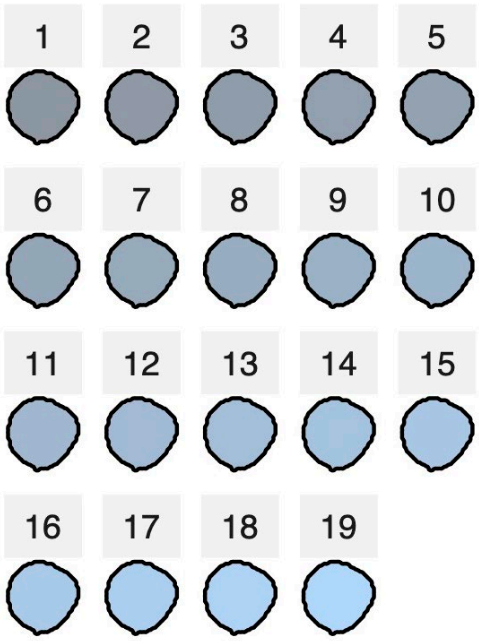

Figure S2. Elliptic fitting process after 19 harmonics (99%). Design: Carlos Rodríguez Rellán.

**UE**	**Accuracy (%)**
10042	65.75
10049	10.53
80	44.74
88	20.27
90	25.22
209	2.89
Overall	34.93

Table S4. Percentage of accuracy in the classification of beads with their respective Stratigraphic Units (UE) achieved by the Linear Discriminant Analysis derived from the GMA data.

UE	Mean	Variance	Standard Deviation	Coefficient of Variation
10042	42.7	171.0	13.1	30.7
10049	35.7	167.0	12.9	36.2
209	31.7	101.0	10.0	31.7
80	29.3	71.2	8.44	28.8
88	30.3	91.9	9.58	31.6

Table S5. Mean, variance, standard deviation and coefficient of variation of the Haralick's circularity of the Montelirio beads according to the Stratigraphic Unit (UE) in which they were found.

UE	Z	P.unadj	P.adj
10042 vs. 10049	4.16848	3.07E-05	4.599609e-04*
10042 vs. 209	14.1091	3.34E-45	5.004969e-44*
10042 vs. 80	20.4687	4.10E-93	6.143914e-92*
10042 vs. 88	17.235	1.45E-66	2.174955e-65*
10042 vs. 90	17.4325	4.67E-68	7.008006e-67*
10049 vs. 209	2.71626	6.60E-03	9.90E-02
10049 vs. 80	4.64953	3.33E-06	4.990385e-05*
10049 vs. 88	3.88189	1.04E-04	1.554722e-03*
10049 vs. 90	3.4246	6.16E-04	9.24E-03
209 vs. 80	3.72699	1.94E-04	2.906665e-03*
209 vs. 88	2.17236	2.98E-02	4.47E-01
209 vs. 90	1.27139	2.04E-01	1.00E+00
80 vs. 88	-1.4658	1.43E-01	1.00E+00
80 vs. 90	-2.7576	5.82E-03	8.74E-02
88 vs. 90	-1.0805	2.80E-01	1.00E+00

Table S6. Bonferroni post-hoc test of Haralick's circularity of the Montelirio beads according to the Stratigraphic Unit (UE) in which they were found (* significant at 0.05).

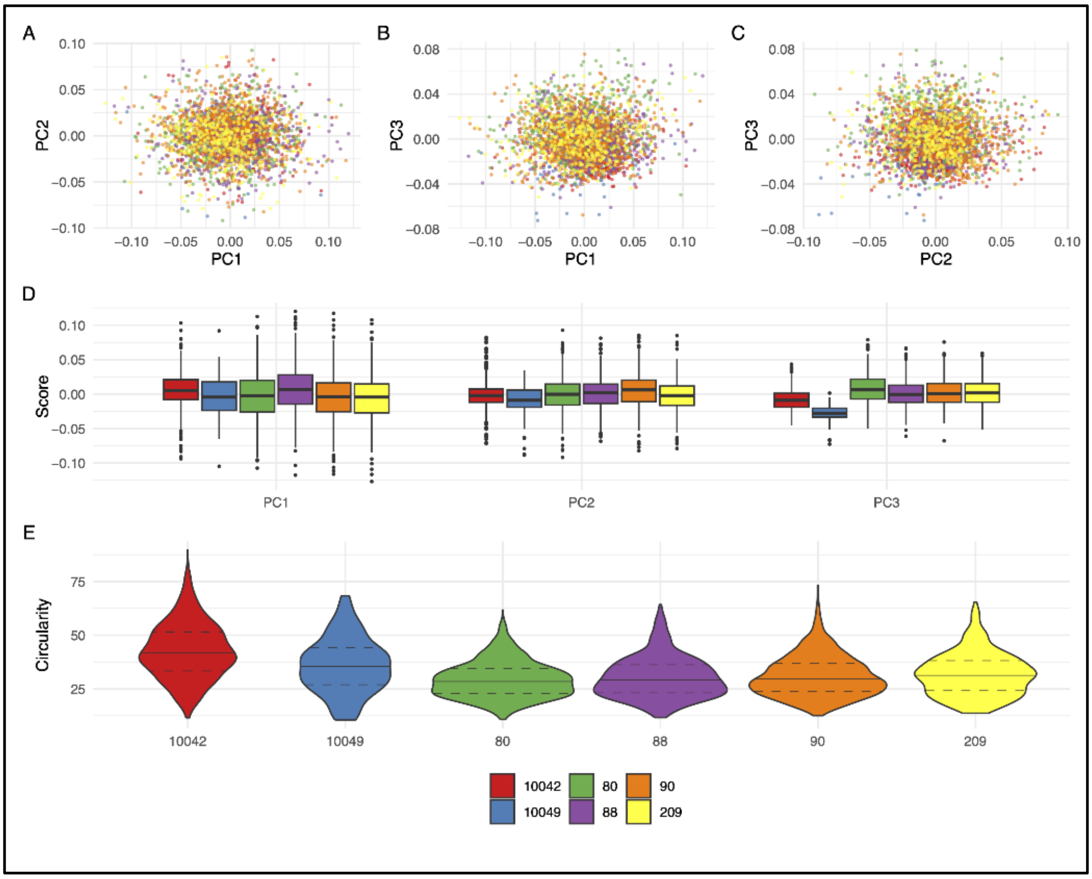

Figure S3: A-C) Principal Component Analysis of the Montelirio beads according to the Stratigraphic Units (UE) within 10.042-10.049 and SC where they were recovered; D) variations between the groups across the first three Principal Components; E) Violin plot displaying the Haralick's circularity of the beads. Results were obtained from a total of 3226 beads, after the outliers that could distort the variability of the set were eliminated. Design: Carlos Rodríguez Rellán.

SUPPLEMENTARY MATERIAL 2. SUMMARY OF RADIOCARBON DATES AND BAYESIAN MODELLING

BURIAL	ID LAB	CONTEXT	MATERIAL	SU	SU INDIVIDUAL	δ13C (‰)	BP	Cal BC 2σ (*)
Montelirio	OxA-41458	MCG-INV6-31	Shell (Pectinidae)	UE167	UE114	-4,1801	5058 ± 19	3555-3191
Montelirio	OxA-41459	MCG-INV1-90	Shell (Cardiidae)	UE344	UE343	1,81452	4819 ± 18	3289-2901
Montelirio	OxA-41319	MCG-INV89-18	Shell (Pectinidae)	UE94	UE102	0,711115	4787 ± 17	3258-2877
Montelirio	OxA-41328	MCG-INV6-91	Shell (Cardiidae)	UE167	UE114	-3,70669	4686 ± 17	3100-2727
Structure 10.049	OxA-42256	PP4-10049-029	Shell (Bivalves)	UE535	Decoration of dagger	0,692905	4597 ± 18	2965-2606
Structure 10.049	OxA-42257	PP4-10049-057	Shell (Bivalves)	UE535	Decoration of dagger	-6,45831	4568 ± 19	2912-2576
Montelirio	OxA-41560	MCG-INV1-60	Shell (Pectinidae)	UE344	UE343	1,10319	4560 ± 19	2906-2574
Montelirio	OxA-41460	MCG-INV1-89	Shell (Cardiidae)	UE344	UE343	-5,66563	4552 ± 18	2900-2567
Montelirio	OxA-41323	MCG-INV8-29	Shell (Pectinidae)	UE95	UE108	-5,44029	4550 ± 17	2896-2566
Montelirio	OxA-41326	MCG-INV356-63	Shell (Pectinidae)	UE357	UE356	-0,858594	4526 ± 17	2881-2542
Montelirio	OxA-41327	MCG-INV356-24	Shell (Cardiidae)	UE357	UE356	1,15406	4501 ± 17	2862-2501
Montelirio	OxA-41322	MCG-INV8-93	Shell (Pectinidae)	UE95	UE108	-5,06905	4486 ± 17	2851-2491
Structure 10.042	OxA-42255	PP4-10042-25	Shell (Pectinidae?)			2,15041	4476 ± 19	2844-2480
Montelirio	OxA-41325	MCG-INV1-61	Shell (Pectinidae)	UE355	UE111	0,970057	4476 ± 17	2843-2476
Montelirio	OxA-41320	MCG-INV93-41	Shell (Cardiidae)	UE94	UE102	-5,96386	4465 ± 17	2834-2469
Montelirio	OxA-41321	MCG-INV93-41	Shell (Cardiidae)	UE94	UE102	-6,6257	4455 ± 17	2834-2462
Montelirio	OxA-41461	MCG-INV1-70	Shell (Cardiidae)	UE344	UE343	-3,43736	4455 ± 18	2833-2461
Montelirio	OxA-41314	MCG-INV93-38	Shell (Cardiidae)	UE94	UE102	1,19113	4457 ± 20	2832-2459
Montelirio	OxA-41313	MCG-INV89-21	Shell (Cardiidae)	UE94	UE102	-5,8561	4450 ± 22	2830-2451
Montelirio	OxA-42253	MCG-INV24-85	Shell (Bivalves)	UE175	Cloth	-6,407	4449 ± 19	2829-2452
Montelirio	OxA-42254	MCG-INV24-27	Shell (Pectinidae)	UE175	Cloth	-6,02412	4437 ± 19	2821-2438
Montelirio	OxA-42252	MCG-INV16-01	Shell (Pectinidae)	UE163	UE103	-5,49186	4432 ± 19	2821-2427
Structure 10.042	OxA-42245	PP4-10042-53	Shell (Pectinidae)			-2,01979	4420 ± 32	2815-2396
Montelirio	OxA-41324	MCG-INV1-58	Shell (Pectinidae)	UE355	UE111	-3,24053	4410 ± 17	2771-2384

Table S7. Radiocarbon dates. (*) Calibrated by Shell Delta_R correction -108 ± 31 14C yr (71). SU: Stratigraphic Unit.

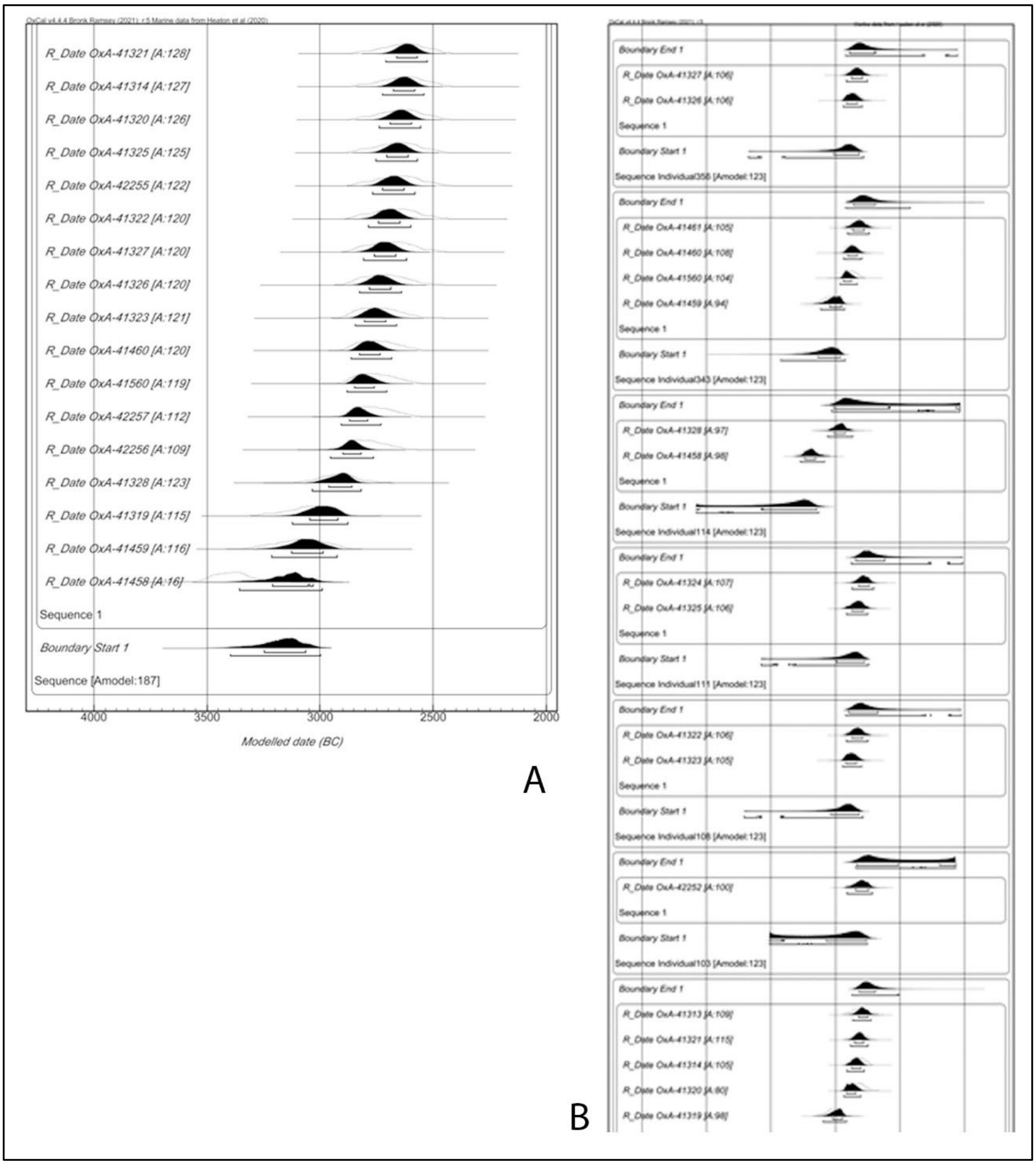

Figure S4: Radiocarbon modelling. A) Bayesian model incorporating all 24 dates on shell bead; B) Bayesian models for beaded attires. Design: Verónica Balsera Nieto.

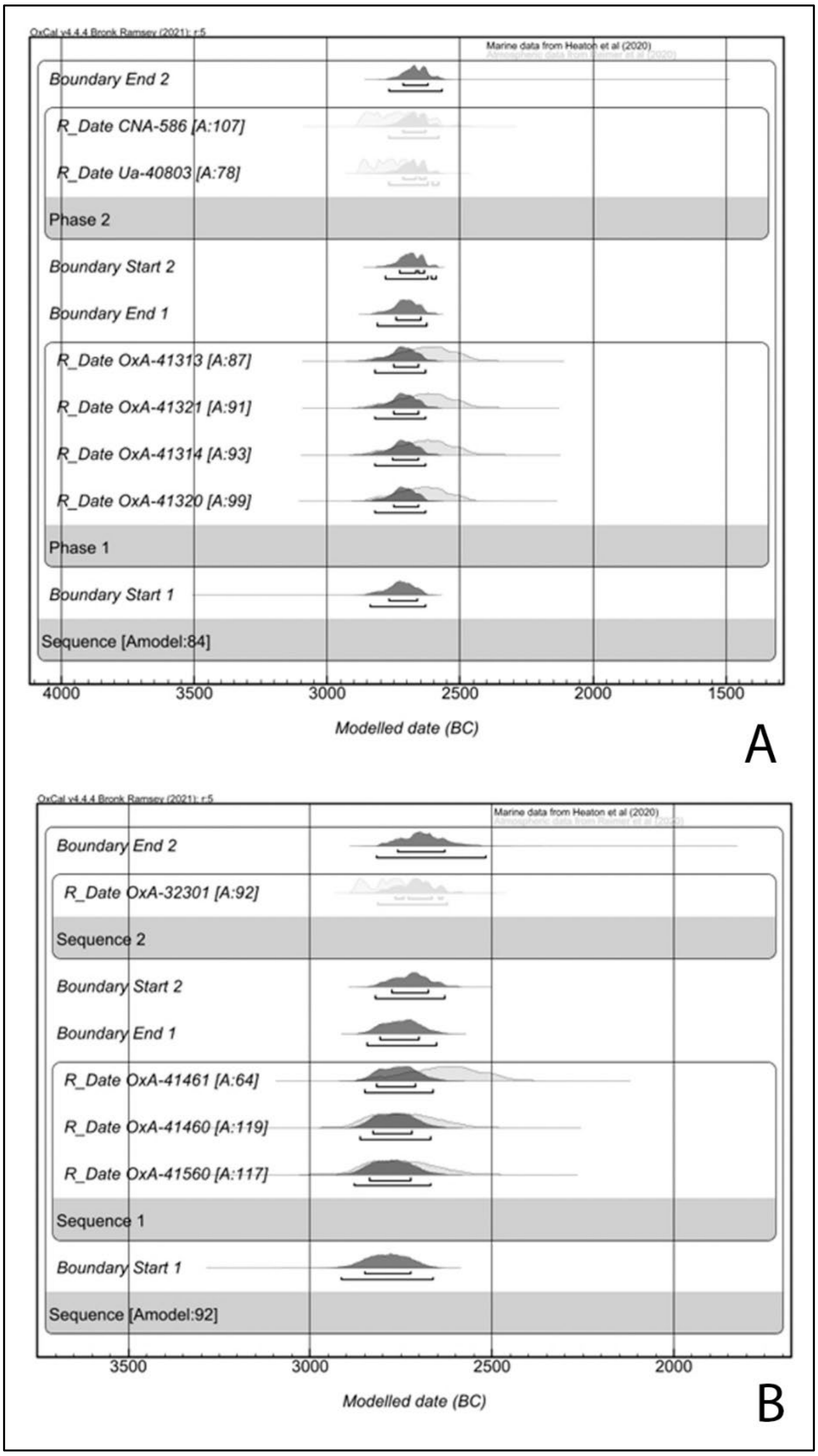

Figure S5: Radiocarbon modelling. A) Two-phase model for Individual UE 102; B) Two-phase model for Individual UE 343. Design: Verónica Balsera Nieto.

Table S8: Bayesian models for Montelirio individuals with dated shell beads

	from	to	SIGMA	from	to	SIGMA
**Boundary End 1**	**-2777**	**-2376**	**1σ**	**-2843**	**-1107**	**2σ**
R_Date OxA-41327	-2747	-2584	1σ	-2827	-2507	2σ
R_Date OxA-41326	-2830	-2667	1σ	-2876	-2586	2σ
Delta_R(-108,31)	-138	-77	1σ	-168	-45	2σ
Curve Marine20						
Sequence 1						
**Boundary Start 1**	**-3029**	**-2632**	**1σ**	**-4346**	**-2569**	**2σ**
			**Sequence Individual356**			

	from	to	SIGMA	from	to	SIGMA
**Boundary End 1**	-2721	-2381	1σ	-2837	-1846	2σ
R_Date OxA-41461	-2731	-2556	1σ	-2811	-2474	2σ
R_Date OxA-41460	-2814	-2666	1σ	-2865	-2593	2σ
R_Date OxA-41560	-2878	-2756	1σ	-2924	-2662	2σ
R_Date OxA-41459	-3088	-2905	1σ	-3227	-2861	2σ
Delta_R(-108,31)	-135	-75.5	1σ	-164.5	-46.5	2σ
Curve Marine20						
Sequence 1						
**Boundary Start 1**	-3267	-2925	1σ	-3850	-2850	2σ
			**Sequence Individual 343**			

	from	to	SIGMA	from	to	SIGMA
**Boundary End 1**	**-3030**	**-1066**	**1σ**	**-3057**	**-1064**	**2σ**
R_Date OxA-41328	-3021	-2847	1σ	-3127	-2736	2σ
R_Date OxA-41458	-3479	-3308	1σ	-3541	-3169	2σ
Delta_R(-108,31)	-145	-79.5	1σ	-175.5	-46	2σ
Curve Marine20						
Sequence 1						
**Boundary Start 1**	**-5160**	**-3295**	**1σ**	**-5164**	**-3259**	**2σ**
			**Sequence Individual 114**			

	from	to	SIGMA	from	to	SIGMA
**Boundary End 1**	**-2679**	**-2233**	**1σ**	**-2750**	**-1029**	**2σ**
R_Date OxA-41324	-2640	-2480	1σ	-2740	-2411	2σ
R_Date OxA-41325	-2739	-2566	1σ	-2832	-2497	2σ
Delta_R(-108,31)	-140.5	-79	1σ	-171	-48	2σ
Curve Marine20						
Sequence 1						
**Boundary Start 1**	**-2993**	**-2548**	**1σ**	**-4154**	**-2488**	**2σ**
			**Sequence Individual 111**			

	from	to	SIGMA	from	to	SIGMA
**Boundary End 1**	**-2787**	**-2345**	**1σ**	**-2839**	**-1053**	**2σ**

R_Date OxA-41322	-2746	-2576	1σ	-2828	-2500	2σ
R_Date OxA-41323	-2839	-2677	1σ	-2887	-2593	2σ
Delta_R(-108,31)	-137.5	-75.5	1σ	-167.5	-45	2σ
Curve Marine20						
Sequence 1						
**Boundary Start 1**	**-3078**	**-2637**	**1σ**	**-4414**	**-2573**	**2σ**
**Sequence Individual 108**						

	from	to	SIGMA	from	to	SIGMA
**Boundary End 1**	**-2671**	**-1139**	**1σ**	**-2685**	**-1139**	**2σ**
R_Date OxA-42252	-2682	-2488	1σ	-2819	-2430	2σ
Delta_R(-108,31)	-140.5	-78.5	1σ	-171	-47	2σ
Curve Marine20						
Sequence 1						
**Boundary Start 1**	**-4024**	**-2514**	**1σ**	**-4024**	**-2505**	**2σ**
**Sequence Individual 103**						

	from	to	SIGMA	from	to	SIGMA
**Boundary End 1**	**-2637**	**-2380**	**1σ**	**-2746**	**-2019**	**2σ**
R_Date OxA-41313	-2648	-2501	1σ	-2729	-2445	2σ
R_Date OxA-41321	-2698	-2562	1σ	-2763	-2495	2σ
R_Date OxA-41314	-2749	-2609	1σ	-2821	-2552	2σ
R_Date OxA-41320	-2835	-2686	1σ	-2868	-2606	2σ
R_Date OxA-41319	-3049	-2886	1σ	-3193	-2827	2σ
Delta_R(-108,31)	-145.5	-86	1σ	-174	-54	2σ
Curve Marine20						
Sequence 1						
**Boundary Start 1**	**-3186**	**-2909**	**1σ**	**-3586**	**-2803**	**2σ**
**Sequence Individual 102**						

SUPPLEMENTARY MATERIAL 3. EXPERIMENTAL WORK.

Estimated time spent making 270.000 beads

Three estimates were made to obtain an approximate understanding of the time spent on the Montelirio beads. Based on a flint-point drilling tool, four estimates were made:

-Estimate #1 as if all the beads had been made from Pecten shell.
-Estimate #2 as if all beads had been made from Cardiidae shell.
-Estimate #3 with a mix (53% Pectinidae, 21% Cardiidae and 26% Indet).
-Estimate #4 with the mean time for a model bead.

First estimate

All the beads are assumed to be made out of Pecten shells, so if it takes 19 minutes to make one Pecten shell bead (see Table S9), it would take 5,130,000 minutes, equivalent to 85,500 hours to make 270,000 Pecten beads. Assuming a working day of 8h/day, 10,687.5 days for one person.

Second estimate

All the beads are assumed to made out of Cardiidae shells, so if it takes 91 minutes to make one Cardiidae shell bead (see Table I), to make 270,000 Cardiidae beads it would take 24,570,000 minutes, equivalent to 409,500 hours. Assuming a work rate of 8 hours/day, that would represent 51,187.5 days for one person.

Third estimate

For this third scenario it would be necessary to know what percentage of these 270,000 beads are from Cardiidae and how many are made using Pectinidae, because they have different processing times due to different shell hardness. This approach is based on a previous study (Ramírez-Cruzado 2020) in which out of 100 examined beads, 53 were made from Pectinidae, 21 from Cardiidae and 26 could not be determined. The time estimate is based on 74% of the 270,000 beads (53 of Pecten + 21 of Cardiidae), for the remaining 26%, the average time obtained with the arithmetic mean for both the other types was used.

53% of 270,000=143,100 beads made from Pecten
21% of 270,000=56,700 beads made from Cardiidae

There would be some 70,200 beads left of unknown shell.
143,100 beads made from Pecten x 19 minutes=2,718,900 minutes
56,700 beads made from Cardiidae x 91 minutes= 5,159,700 minutes
2,718,000 + 5,159,700= 7,877,700 minutes= 131,295 hours to manufacture 74% of the Montelirio beads.
-Time taken for each bead made from Pecten=19min.
-Time taken for each bead made from Cardiidae=91min.
-The time invested for a typical bead would be the average of the time taken for each type: (19+91)/2=55 minutes.
Therefore, the 70,200 beads left will be calculated on the basis of the time obtained for a model bead.

70,200 x 55 minutes= 3.861.000 minutes
7,877,700 +3.887.000 =11,738,700 minutes for the 100% of the beads, equivalent to 195,645 hours corresponding to 24,455.625 days (8-hour working day).

Fourth estimate

An average time of 55 minutes per bead is assumed for the 270,000 beads. To make 270,000 beads would have taken 14,850,000 minutes equivalent to 247,500 hours.

Real time assessment

Table S9 summarises the real times as observed in four different experiments with the beads:

The estimated time to make a Pecten bead is the mean of the total times of experiments #1 (14 minutes) and #3 (23 minutes), the mean time being 19 minutes. The same is true for the beads made with Cardiidae, the processing time is the average of the times of experiments #2 (98 minutes) and #4 (85 minutes), with a mean time of 91 minutes.

Experiment #	Shell Type	Rock Type	Task	Time
#1	Pectinidae	Sandstone	edge abrasion (shaping)	6:24 min
	Pectinidae	Flint	perforation	8 min
#2	Cardiidae	Sandstone	edge abrasion (shaping)	21:55 min
	Cardiidae	Flint	perforation	1h 16 min
#3	Pectinidae	Sandstone	edge abrasion (shaping)	7 min
	Pectinidae	Flint	perforation	16 min
#4	Cardiidae	Sandstone	edge abrasion (shaping)	15 min
	Cardiidae	Flint	perforation	1 h 10 min

Table S9. Experimental work. Real time invested in the elaboration of a bead

Table S10 summarises the times estimated for each scenarios:

Scenario/Time invested	Pecten	Cardiidae	74% of the beads	100% of the beads	Ideal beads
Time invested scenario 1	5,130,000 min/ 85,500 h	-	-	-	-
Time invested scenario 2	-	24,570,000 min/ 409,500 h	-	-	-
Time invested scenario 3	-	-	7,877,700 min/131,295 h	11,738,700 min/ 195,645 h	-
Time invested scenario 4	-	-	-	-	14,850,000 min/ 247,500 h

Table S10. Summary of the various scenarios contemplated in the experimental work

If we assume that a Copper Age craftperson was at least 5 times faster:

Scenario/Time invested	Pecten	Cardiidae	74% of the beads	100% of the beads	Ideal beads
Time invested scenario 1	1.026.000 min/ 17.100 h	-	-	-	-
Time invested scenario 2	-	4.914.000 min/81.900 h	-	-	-
Time invested scenario 3	-	-	1.575.540 min/26,259 h	2,347,740 min/ 39.129h	-
Time invested scenario 4	-	-	-	-	2,970,000 min/49,500h

Table S11. Summary of the various scenarios contemplated in the experimental work as corrected according to hypothetical Copper Age craftperson skill

Estimates concerning the number of shells involved

In order to estimate the overall number of shells involved in the production of the whole Montelirio bead assemblage, an average Pecten shell was chosen. In order to calculate the total area (At), the shell was divided into two geometric figures, a circle and a trapeze. Later, the preform shown in Figure S6 A2, a rectangle of 3.3 x 2.2 cm was taken as reference. Finally, the area of the shell was divided by the area of the preform, in order to work out how many typical preforms would a typical Pecten shell yield.

$A_T=A_1+A_2$
A_1= circle area – circle segment area*
A_2= trapeze area
*the circle segment is the area of the circle occupied by the trapeze, and therefore is has to be subtracted to the circle area.
A_1 (circle area)- (circle segment area) = $(\pi 6^2)$ - $[\frac{\pi 6^2 \cdot 106{,}26}{360} - \frac{8 \cdot 3}{2}]$=91,715 cm^2
A_2 (trapeze area) =$\frac{(8+4)\cdot 3}{2}$= 18 cm^2
$A_T=A_1+A_2$= 91,715+18= 109,715 cm^2

The total area of the selected Pecten shells is 109,715 cm^2

The area of the selected preform is:

$Apreform = b \cdot h$= 3,3 x 2,2 = 7, 26 cm^2

In order to work out how many preforms can be obtained from the shell of the size of the example selected:

$\frac{Atotal}{Apreform}$= $\frac{109{,}715}{7{,}26}$ = 15,11 preforms ≈ 15 preforms

Therefore, a standard shell would produce 15 bead preforms.

Discussion

As indicated in the introduction to the paper, bead making is a well recorded archaeological phenomenon and widespread in time and space, whether for use as exchange currency in the case of the Chumash (41-43) or as ornaments and clothing (12, 35).
Here we compare the production of these beads with others of a similar typology found in other archaeological contexts, which will give us a broader context for the production of discoidal beads and of the magnitude of the complete set of beads from the Montelirio tholos, some 260,000. It is interesting to note that in the literature reviewed there is no experimental archaeology or estimation of the time taken to produce accounts for the Iberian Peninsula, so this paper is the first of its kind. Of all the studies on bead finds mentioned in this paper, only those from North America contain an approximation of the time taken to produce the beads. For example, in the case of Mound 72 at Cahokia, estimates range from 20000-40000 beads at most (12, 45), a much lower number than those found at Montelirio, taking into account that the North American site is larger (about 890 hectares) than the Valencina mega-site site the Montelirio tholos is part of. In terms of time spent, the estimate for Cahokia Mound 72 was 45 minutes for a discoidal bead, with a total of 9024 working hours to produce 12,000 beads (45). The shell type used to make these beads is not known. If we compare it with the time for a model bead from Estimate 4, being 57.5 minutes, the time spent would be longer for those from Montelirio. We must also be aware that the beads from Montelirio are smaller, which means that more work is involved. Be that as it may, the large number of beads found at Montelirio means that any estimate of the time spent would provide larger figures.

There is a peculiarity regarding the context in which the Montelirio beads were found, and that is the absence of a bead-making area. At other sites where a large number of beads have been identified, traces of bead making have also been found. In other words, associated with the beads, what could be the bead processing zone has been identified, and sometimes, in the same stratigraphic levels, the presence of the remains of perforators or fragmented beads has been recorded (45, 78, 79). These areas would be characterised by the finding of waste material resulting from processing, such as fragments of beads and flint flakes, remains of sandstone used for polishing, tools, finished or half-finished beads, storage areas for raw materials. The finding of bead workshops provides two insights: 1) unequivocal relationship of shell drilling with lithic tools and the creation of specific tools for this work, 2) the beads were made in the same place and at the same time as they were used, or at least part of the production.

In the Valencina, the absence of evidence of this type of workshop-zone, at least to date, is interesting considering the large number of beads identified in Montelirio. This fact could indicate that the communities that made use of the Montelirio´s tholos were already receiving the final product and would only have to carry out the last necessary steps such as crimping for use as clothing or offerings.

Another aspect to discuss is the existence of some kind of specialisation in the production of this jewelry. In this sense, Kozuch (45) stresses that it is necessary to differentiate between standardisation and specialisation, while the former would be the production of many products following certain canons, the latter would be a full-time activity that would exclude the performance of other activities, in exchange for the goods necessary to live. Specialisation can in turn be divided into those who choose the raw materials, those who make the tools, those who carry out the different stages such as fragmentation, drilling, polishing the beads, etc.

Bearing this in mind, we can say that the Montelirio beads have a high degree of standardization, as the vast majority have the same size and thickness, as well as a meticulously cared-for circular shape. What cannot be inferred is the presence of specialized full-time craftspeople in the Chalcolithic community of Valencina, as there is no evidence of workshops. But it is clear that whoever made them were very skilled craftspeople, regardless of whether they worked full-time or not.

As all the beads display similar measurements (see Data S1-3) it has not been possible to differentiate between different standards in the bead patterns. The use of the beads could be restricted according to the social group, as in this case beads have only been detected making garments in funerary contexts.

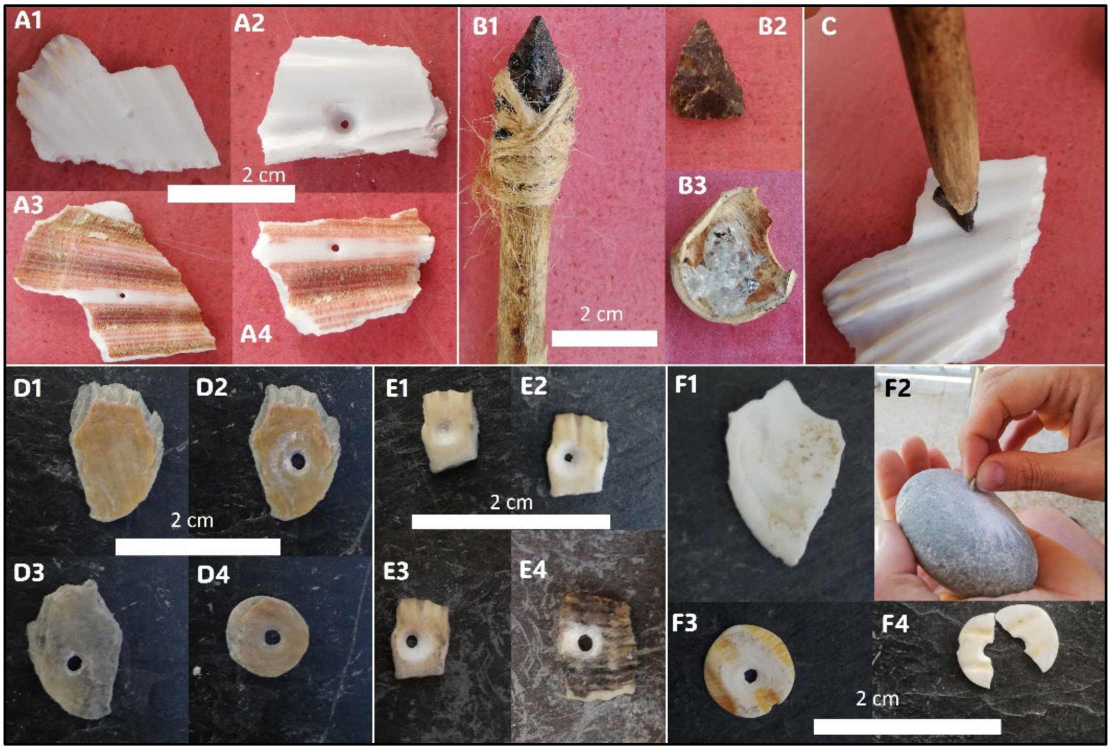

Figure S6: Experimental work with discoidal beads on marine shell. A: extraction of the base-form and perforation on shell of *Pectinidae*; A1-2: internal face of the shell; A3-4: external face of the shell; B: tools used in the process; B1: flint drill; B2: detail of the knapped tip of the flint drill; B3: hazelnut shell with pine resin; C: drilling of the shell; D: phases in the process of regularisation of the bead on shell of *Pectinidae*; D1: base-form; D2: drill on the external face of the base-form; D3: drill on the internal face of the base-form; D4: rounding of the edges through abrasion; E: phases in the perforation process of the bead on shell of *Cardiidae*; E1: base-form; E2: start of the perforation on the internal face of the base-form; E3: perforation on the internal face of the base-form; E4: external face of the perforated base-form; F: phases in the regularisation process of the bead on shell of *Cardiidae*; F1: base-form; F2: abrasion of the edges of the base-form with a pebble; F3: finished bead; F4: breakage of the finished bead. Photographs: Samuel Ramírez-Cruzado.

Other Supplementary Materials for this manuscript include the following:

Data S1: Morphometric data for Montelirio Large Chamber
Data S2: Morphometric data for Montelirio Small Chamber
Data S3: Morphometric data for Structure 10.042-10.049
Data S4: Full data of all radiocarbon dates available for Montelirio and Structure 10.042-10.049 (marine shell and human bone)

REFERENCES AND NOTES

1. A. Fernández Flores, L. García Sanjuán, M. Díaz-Zorita, Eds. *Montelirio: Un Gran Monumento Megalítico de la Edad del Cobre* (Junta de Andalucía, 2016). [Montelirio: A Great Copper Age Megalithic Monument].

2. L. García Sanjuán, J. M. Vargas Jiménez, L. M. Cáceres Puro, M. E. Costa Caramé, M. Díaz-Guardamino Uribe, M. Díaz-Zorita Bonilla, A. Fernández Flores, V. Hurtado Pérez, P. M. López Aldana, E. Méndez Izquierdo, A. Pajuelo Pando, J. Rodríguez Vidal, D. W. Wheatley, A. Delgado-Huertas, E. Dunbar, A. Mora González, C. Bronk Ramsey, A. Bayliss, N. Beavan, D. Hamilton, A. Whittle, Assembling the dead, gathering the living: Radiocarbon dating and Bayesian modelling for Copper Age Valencina de la Concepción (Sevilla, Spain). *J. World Prehist.* **31**, 179–313 (2018).

3. L. G. Sanjuán, M. L. Triviño, M. Cintas-Peña, Ivory, elites and lineages in Copper Age Iberia. Exploring the wider significance of the Montelirio tomb. *Madrider Mitteilungen* **59**, 23–65 (2018).

4. L. García Sanjuán, M. Cintas-Peña, M. Díaz-Guardamino, J. Escudero Carrillo, M. Luciañez Triviño, C. Mora Molina, S. Robles Carrasco, "Burial practices and social hierarchisation in Copper Age Southern Spain: Analysing tomb 10.042–10.049 of Valencina de la Concepción (Seville, Spain)" in *Megaliths, Societies, Landscapes. Early Monumentality and Social Differentiation in Neolithic Europe*, Müller, J.; Hinz, M. and Wunderlich, M, Eds. (Frühe Monumentalität und soziale Differenzierung 18/III, 2019), pp. 1005–1037.

5. L. García Sanjuán, R. Montero Artús, S. Emslie, J. A. Lozano Rodríguez, M. Luciañez Triviño, Beautiful, magic, lethal: A social perspective of cinnabar use and mercury exposure at the Valencina Copper Age mega-site (Spain). *J. Archaeol. Method Theory* **31**, 1006–1061 (2024).

6. M. Luciañez Triviño, L. García Sanjuán, T. Schuhmacher, Crafting idiosyncrasies. Early social complexity, ivory and identity-making in Copper Age Iberia. *Camb. Archaeol. J.* **32**, 23–60 (2021).

7. M. Cintas-Peña, M. Luciañez-Triviño, R. Montero Artús, A. Bileck, P. Bortel, F. Kanz, K. Rebay-Salisbury, L. García Sanjuán, Amelogenin peptide analyses reveal female leadership in Copper Age Iberia (c. 2900-2650 BC). *Sci. Rep.* **13**, 9594 (2023).

8. L. García Sanjuán, C. Scarre, D. W. Wheatley, The mega-site of Valencina de la Concepción (Seville, Spain): Debating settlement form, monumentality and aggregation in southern Iberian Copper Age societies. *J. World Prehist.* **30**, 239–257 (2017).

9. J. Guilaine, Siret's Smile. *Antiquity* **92**, 1247–1259 (2018).

10. A. Whittle, *Times of Their Lives. Hunting History in the Archaeology of Neolithic Europe* (Oxbow Books, 2017).

11. B. Gaydarska, J. Chapman, *Megasites in Prehistoric Europe. Where Strangers and Kinsfolk Met* (Cambridge Univ. Press, 2022).

12. M. Díaz-Guardamino, D. W. Wheatley, E. F. Williams, J. A. Garrido Cordero, "Los textiles elaborados con cuentas perforadas de Montelirio" in *Montelirio: Un Gran Monumento Megalítico de la Edad del Cobre*, Seville, A. Fernández Flores, L. García Sanjuán, M. Díaz-Zorita Bonilla, Eds. (Junta de Andalucía, 2016), chap. 14, pp. 345–365. [Montelirio's textiles made with perforated beads].

13. J. C. Pecero Espín, "Caracterización antropológica de los restos óseos humanos del tholos del Montelirio" in *Montelirio: Un Gran Monumento Megalítico de la Edad del Cobre, Sevilla*, A. Fernández Flores, L. García Sanjuán, M. Díaz-Zorita Bonilla, Eds. (Junta de Andalucía, 2016), chap. 16, pp. 409–442. [Anthropological characterization of human skeletal remains from the Montelirio tholos].

14. A. R. Brand, "Scallop ecology: distributions and behaviour" in *Developments in aquaculture and fisheries science* (Elsevier, 2006), pp. 651–744, vol. 35.

15. D. Minchin, Introductions: Some biological and ecological characteristics of scallops. *Aqua. Living Res.* **16**, 521–532 (2003).

16. M. M. Rufino, M. B. Gaspar, A. M. Pereira, F. Maynou, C. C. Monteiro, Ecology of megabenthic bivalve communities from sandy beaches on the south coast of Portugal. *Sci. Mar.* **74**, 163–178 (2010).

17. M. Peharda, D. Ezgeta-Balić, M. Radman, N. Sinkjević, N. Vrgoč, I. Isajlović, Age, growth and population structure of Acanthocardia tuberculata (Bivalvia: Cardiidae) in the eastern Adriatic Sea. *Scientia Marina* **76**, 59–66 (2012).

18. A. Rharrass, M. Talbaoui, M. Gaspar, M. Kabine, N. Rharbi, Gametogenic cycle of the rough cockle Acanthocardia tuberculata (Mollusca: Bivalvia) in the M'diq Bay (SW Mediterranean Sea). *Sci. Mar.* **80**, 359–368 (2016).

19. S. K. Malham, T. H. Hutchinson, M. Longshaw, A review of the biology of European cockles (Cerastoderma spp.). *J. Mar. Biol. Assoc. United Kingdom* **92**, 1563–1577 (2012).

20. J. P. Bard, "Le Metamorphisme Regional Progressif des Sierras d'Aracena en Andalousie Occidentale (Espagne). Sa Place dans le Segment Hercynien Sub-Iberique," thesis, Montpellier (1969). [The Regional Progressive Metamorphism of the Sierras de Aracena in Western Andalusia (Spain). Its place in the Sub-Iberian Hercynian Segment].

21. C. Ponce, J. F. Simancas, A. Azor, D. J. Martínez Poyatos, G. Booth-Rea, I. Expósito, Metamorphism and kinematics of the early deformation in the Variscan suture of SW Iberia. *J. Metam. Geol.* **30**, 625–638 (2012).

22. IGME (2015): *Mapa Geológico de España. Escala 1:200.000. Sevilla–Puebla de Guzmán* [Geological and Mining Institute of Spain (IGME), 2015]. [Geological Map of Spain. Scale 1:200.000. Sevilla-Puebla de Guzmán]

23. R. M. Haralick, A measure for circularity of digital figures. *IEEE Trans. Syst. Man Cybern.* **SMC-4**, 394–396 (1974).

24. L. García Sanjuán, A. Fernández Flores, M. Díaz-Zorita Bonilla, "Montelirio. Valoración e interpretación de una tumba excepcional" in *Montelirio: Un Gran Monumento Megalítico de la Edad del Cobre*, Seville, A. Fernández Flores, L. García Sanjuán, M. Díaz-Zorita Bonilla,

Eds. (Junta de Andalucía, 2016), chap. 22, pp. 503–553. [Montelirio. Assessment and interpretation of an exceptional tomb].

25. A. Pinilla, M. A. Bustillo, Silicofitolitos en secuencias arcillosas con silcretas. Mioceno Medio, Madrid. *Monografías del Centro de Ciencias Medioambientales* **4**, 255–265 (1997). [Silicophytoliths in clay sequences with silcretes].

26. J. A. Afonso Vargas, "Aplicación del Análisis de Fitolitos y otros Microfósiles al Estudio de Yacimientos, Materiales Arqueológicos y Edáficos de las Islas Canarias. Los Ejemplos de Las Cañadas del Teide (Tenerife), La Cerera (Arucas, Gran Canaria) y Otras Zonas de Aplicación Experimental", thesis, University of La Laguna, Santa Cruz de Tenerife, Spain (2014). [Application of the Analysis of Phytoliths and other Microfossils to the Study of Archaeological and Edaphic Materials of the Canary Islands. The Examples of Las Cañadas del Teide (Tenerife), La Cerera (Arucas, Gran Canaria) and Other Areas of Experimental Application].

27. D. Piperno, *Phytoliths, A Comprehensive Guide for Archaeologists and Paleoecologists* (AltaMira Press, 2006).

28. E. Kvavadze, O. Bar-Yosef, A. Belfer-Cohen, E. Boaretto, N. Jakeli, Z. Matskevich, T. Meshveliani, 30,000-year-old wild flax fibers. *Science* **325**, 1359–1359 (2009).

29. C. Alfaro-Giner, Spain in *Textiles and Textile Production in Europe from Prehistory to AD 400*, M. Gleba, U. Mannering, Eds. (Oxbow Books, 2012), pp. 334–346.

30. F. Molina González, M. O. Rodríguez Ariza, S. Jiménez-Brobeil, M. Botella, La Sepultura 121 del yacimiento argárico de El Castellón Alto (Galera, Granada). *Trabajos de Prehistoria* **60**, 153–158 (2003). [Burial 121 of the Argaric site of El Castellón Alto].

31. D. Bar Yosef Mayer, C. Bonsall, A. M. Choyke, Eds., *Not Just for Show. The Archaeology of Beads, Beadwork and Personal Ornaments* (Oxbow Books, 2017).

32. K. Karklins, *Researching The World's Beads. An Annotated Bibliography* (Society of Bead Researchers, 2016).

33. K. Karklins, *Researching The World's Beads. An Annotated Bibliography. Archaeometric Analysis* (Society of Bead Researchers, 2024).

34. E. Trinkaus, A. P. Buzhilova, Diversity and differential disposal of the dead at Sunghir. *Antiquity* **92**, 7–21 (2018).

35. L. Lbova, The Siberian Paleolithic site of Mal'ta: A unique source for the study of childhood archaeology. *Evol. Hum. Sci.* **3**, e9 (2021).

36. H. Alarashi, M. Benz, J. Gresky, A. Burkhardt, A. Fischer, L. Gourichon, M. Gerlitzki, M. Manfred, J. Sakalauskaite, B. Demarchi, M. Mackie, M. Collins, C. Odriozola, J. A. Garrido Cordero, M. A. Avilés, L. Vigorelli, A. Re, H. G. K. Gebel, Threads of memory: Reviving the ornament of a dead child at the Neolithic village of Ba`ja (Jordan). *PLOS ONE* **18**, e0288075 (2023).

37. J. L. Pascual Benito, "Los talleres de cuentas de Cardium en el Neolítico peninsular" in *Actas III Congreso de Neolítico en la Península Ibérica (Santander, 5–8 octubre de 2003)*, P. Arias Cabal, R. Ontañón Peredo, C. García-Moncó Piñeiro, Eds. (Monografías del Instituto Nacional de Investigaciones Prehistóricas de Cantabria, 2005), pp. 227–286. [The bead workshops of Cardium in the peninsular Neolithic period].

38. M. I. Dias, Z. Kasztovszky, M. I. Prudêncio, I. Harsányi, I. Kovács, Z. Szőkefalvi-Nagy, J. Mihály, G. Káli, A. C. Valera, A. L. Rodrigues, Investigating beads from Chalcolithic funerary cremation contexts of Perdigões, Portugal. *J. Archaeol. Sci.* **20**, 434–442 (2018).

39. M. Oliva Poveda, Els ornaments personals de la primera meitat del segon mil·lenni ane del jaciment de Can Roqueta-II (Est) , Sabadell. *Cypsela* **15**, 229–249 (2004). [Personal ornaments from the first half of the second millennium BCE from the site of Can Roqueta-II (east), Sabadell].

40. M. Fauvelle, Mobile Mounds: Asymmetrical exchange and the role of the Tomol in the development of Chumash complexity. *California Archaeol.* **3**, 141–158 (2011).

41. M. Fauvelle, *Shell Money. A Comparative Study* (Cambridge Univ. Press, 2024).

42. L. H. Gamble, The origin and use of shell bead money in California. *J. Anthropol. Archaeol.* **60**, 101237 (2020).

43. M. L. Fowler, J. Rose, B. Vander Leest, S. R. Aler, *The Mound 72 Area: Dedicated and Sacred Space in Early Cahokia* (Illinois State Museum Reports of Investigations no. 54, Santa Fe, Ancient City Press, 1997).

44. L. Kozuch, Shell bead crafting at Greater Cahokia. *North American Archaeologist* **43**, 64–94 (2022).

45. A. Baadsgaard, All the Queen's Clothes: Identifying Female Royalty at Early Dynastic Ur (Near Eastern Archaeology, 2016), vol. 79, pp. 148–155.

46. P. Shukla, *Costume: Performing Identities through Dress* (Indiana Univ. Press, 2015).

47. J. Butler, *Gender Trouble. Feminism and the Subversion of Identity* (Nueva York, 1999).

48. E. Robertson Martinez, "Social Representations and Women Who Live as Men in Northern Albania" thesis, University of Cambridge, England (2020).

49. M. Diniz, "Pesos de tear e tecelagem no Calcolitico em Portugal", *Actas do 1er Congresso de Arqueologia Peninsular (Porto, 12–18 de Outubro de 1993)*. 4, 133–146 (1994). [Weights for looms and weaving in the Chalcolithic period in Portugal].

50. L. M. C. Rollán, Las manufacturas textiles en la Prehistoria: Las placas de telar en el Calcolítico peninsular. *Zephyrus* **49**, 125–145 (1996). [Textile Manufactures in Prehistory: The loom plates in the peninsular Chalcolithic period].

51. M. Murillo-Barroso, "El ámbar del tholos de Montelirio" in *Montelirio: Un Gran Monumento Megalítico de la Edad del Cobre, Sevilla*, A. Fernández Flores, L. García Sanjuán, M. Díaz-Zorita Bonilla, Eds. (Junta de Andalucía, 2016), chap. 13, pp. 311–344. [Amber from the Montelirio tholos].

52. M. Luciañez Triviño, L. García Sanjuán, "Los marfiles del tholos de Montelirio" in *Montelirio: Un Gran Monumento Megalítico de la Edad del Cobre, Sevilla*, A. Fernández

Flores, L. García Sanjuán, M. Díaz-Zorita Bonilla, Eds. (Junta de Andalucía, 2016), chap. 10, pp. 245–272. [The ivories of the Montelirio tholos].

53. K. Grömer, H. Rösel-Mautendorfer, L. B. Jørgensen, Visions of dress: Recreating Bronze Age clothing from the Danubian region. *Textile J. Cloth Cult.* **11**, 218–241 (2013).

54. J. Gulizio, Textiles for the Gods? Linear B evidence for the use of textiles in religious ceremonies in *Kosmos: Jewellery, Adornment and Textiles in the Aegean Bronze Age. Proceedings of the 13th International Aegean Conference/13e Rencontre Égéenne Internationale, University of Copenhagen, Danish National Research Foundation's Centre for Textile Research, 21–26 April 2010*, M-L. Nosch, R. Laffineur, Eds. (Aegaeum 33 series, 2012), pp. 279–285.

55. M. L. Sørensen, Reading dress: The construction of social categories and identities in Bronze Age Europe. *J. European Archaeol.* **5**, 93–114 (1997).

56. J. Schneider, The anthropology of cloth. *Ann. Rev. Anthropol.* **16**, 409–448 (1987).

57. J. Schneider, A. B. Weiner, Cloth and the organization of human experience. *Curr. Anthropol.* **27**, 178–184 (1986).

58. L. M. Cáceres-Puro, F. Muñiz Guinea, J. Rodríguez Vidal, J. M. Vargas, T. Donaire, Marine bioerosion in rocks of the prehistoric *tholos of La Pastora* (Valencina de la Concepción, Seville, Spain): Archaeological and palaeoenvironmental implications. *J. Archaeol. Sci.* **41**, 435–446 (2014).

59. L. M. Cáceres, J. M. Vargas, F. Muñiz, T. Donaire, L. García Sanjuán, C. Odriozola, J. Rodríguez-Vidal, Natural "megalithic art" at Valencina (Seville): A geoarchaeological approach to stone, architecture, and cultural choice in Copper Age Iberia. *Archaeol. Anthropol. Sci.* **11**, 4621–4641 (2019).

60. C. L. von Lettow-Vorbeck, M. T. Aparicio Alonso, R. Araujo, L. Llorente-Rodriguez, A. Morales-Muñiz, La fauna del Sector PP4-Montelirio del yacimiento prehistórico de Valencina de la Concepción (Sevilla). Economía y simbolismo de los animales en una

comunidad del III milenio. *Menga Revista de Prehistoria de Andalucía* **4**, 69–102 (2014). [The fauna of the PP4-Montelirio Sector of the prehistoric site of Valencina de la Concepción].

61. J. Chapman, B. Gaydarska, V. Slavchev, "The life histories of Spondylus shell rings from the Varna I Eneolithic cemetery (north-east Bulgaria): transformation, revelation, fragmentation and deposition", in VVAA: *The Varna Eneolithic Necropolis and Problems of Prehistory in Southeast Europe*, V. Slavchev, Ed. (Acta Musei Varnaensis, 2008), pp. 139–162.

62. F. Ifantidis, M. Nikolaidou, Eds., *Spondylus in Prehistory: New Data and Approaches. Contributions to the Archaeology of Shell Technologies* (British Archaeological Reports, International Series 2216, Archaeopress, 2011).

63. J. Laver, "The Cradle of Venus" in *The Scallop: Studies of a Shell and its Influences on Humankind*, I. Cox, Ed. (Shell, 1957), pp. 73–89.

64. C. Hobler, "The Badge of St James" in *The Scallop: Studies of a Shell and its Influences on Humankind*, I. Cox, Ed. (Shell, 1957), pp. 49–72.

65. A. Jones, Local colour: Megalithic architecture and colour symbolism in Neolithic Arran. *Oxf. J. Archaeol.* **18**, 339–350 (1999).

66. T. Earle, M. Spriggs, Political economy in prehistory: A Marxist approach to pacific sequences. *Curr. Anthropol.* **56**, 515–544 (2015).

67. M. Helms, *Ancient Panama: Chiefs in Search of Power* (Texas Press, 1976).

68. C. S. Hoggard, J. McNabb, J. Cole, The application of elliptic Fourier analysis in understanding biface shape and symmetry through the British Acheulean. *J. Paleo. Arch.* **2**, 115–133 (2019).

69. L. Timbrell, P. de la Peña, A. Way, C. Hoggard, L. Backwell, F. d'Errico, L. Wadley, M. Grove, Technological and geometric morphometric analysis of 'post-Howiesons Poort points' from Border Cave, KwaZulu-Natal, South Africa. *Quaternary Sci. Rev*, **297**, 107813 (2022).

70. A. Bayliss, N. Beavan, C. Bronk Ramsey, A. Delgado-Huertas, M. Díaz-Zorita Bonilla, E. Dunbar, "La cronología radiocarbónica del tholos de Montelirio" in *Montelirio: Un Gran Monumento Megalítico de la Edad del Cobre, Seville*, A. Fernández Flores, L. García Sanjuán, M. Díaz-Zorita Bonilla, Eds. (Junta de Andalucía, 2016), chap.21, pp. 485–502. [The radiocarbon chronology of the Montelirio tholos].

71. J. M. M. Martins, A. M. M. Soares, Marine radiocarbon reservoir effect in Southern Atlantic Iberian coast. *Radiocarbon* **55**, 1123–1134 (2013).

72. C. R. Arbelo Rodríguez, J. A. Afonso Vargas, A. Rodríguez Rodríguez, Estudio preliminar de fitolitos en suelos agrícolas de Tenerife (Islas Canarias). Cereales y otros grupos vegetales in *Retos y Oportunidades en la Ciencia del Suelo. Actas del VI Congreso Ibérico de la Ciencia del Suelo (Santiago de Compostela 22–25 de junio de 2014)*, F. Macías Vázquez, M. Díaz Raviña, M. T. Barral Silva, Eds. (Andavira, 2014), pp. 37–40. [Preliminary study of phytoliths in agricultural soils of Tenerife (Canary Islands). Cereals and other plant groups].

73. J. Afonso-Vargas, I. La Serna-Ramos, M. Arnay-de-la-Rosa, Fungal spores located in 18th century human dental calculi in the church "La Concepción" (Tenerife, Canary Islands). *J. Archaeol. Sci. Rep.* **2**, 106–113 (2015).

74. S. Ramírez-Cruzado, "Las Cuentas Perforadas del Tholos de Montelirio (Castilleja de Guzmán, Sevilla). Una Aproximación Comparativa, Geoarqueológica y Experimental," thesis, University of Sevilla, Sevilla, Spain (2020). [The Perforated Beads from the Montelirio tholos (Castilleja de Guzmán, Sevilla). A Comparative, Geoarchaeological and Experimental Approach].

75. B. Malinowski, *Argonauts of the Western Pacific: An Account of Native Enterprise and Adventure in the Archipelagoes of Melanesian New Guinea* (Routledge, 1922).

76. S. Leonardt, "Artifactos Malacológicos en el Bosque y Ecotono Bosque-Estepa del Noroeste de Patagonia," thesis University of Buenos Aires, Argentina (2013). [Malacological Artifacts in the Forest and Forest-Steppe Ecotone of Northwest Patagonia].

77. S. Leonardt, La elaboración de cuentas con valvas de moluscos en Patagonia a través de la arqueología experimental. *Comechingonia* **23**, 279–302 (2019). [The elaboration of beads with mollusk shells in Patagonia through experimental archaeology].

78. C. Perlès, P. Pion, The Cerastoderma bead production at Franchthi (Greece): A case of apprenticeship? in *Beauty and the Eye of the Beholder: Personal Adornments Across the Millenia*., M. Mărgărit, A. E. Boroneanţ, Eds. (Editura Cetatea de Scaun, 2020).

79. M. Guinea, Un sistema de producción artesanal de cuentas de concha en un contexto doméstico manteño: Japoto (provincia de Manabí, Ecuador). *Bulletin de l'Institut français d'études andines* **35**, 299–312 (2006). [An artisanal production system of shell beads in a manteño domestic context: Japoto].
